# Correlates of mobile device use in young children: a systematic review and meta-analysis

**DOI:** 10.1136/bmjph-2025-004305

**Published:** 2026-06-17

**Authors:** Liane B de Azevedo, Dan Jones, Colette Marr, Amy Hughes, Elizabeth Goyder, Mark Clowes, Emily Pulsford, John Stephenson

**Affiliations:** 1 School of Sport & Physical Activity, College of Health, Wellbeing and Life Sciences, Sheffield Hallam University, Sheffield, UK; 2 School of Health and Life Sciences, Teesside University, Middlesbrough, UK; 3 School of Health and Related Research, The University of Sheffield, Sheffield, UK; 4 School of Human and Health Sciences, University of Huddersfield, Huddersfield, UK

**Keywords:** Public Health, Systematic Review, Digital Technology

## Abstract

**Introduction:**

Mobile device use has increased in recent years among young children. This systematic review and meta-analysis aims to identify and quantify the correlates of mobile device use (tablets and smartphones) and problematic smartphone use (PSU) in young children.

**Methods:**

The following databases were searched up to March 2026: MEDLINE, Scopus, Embase, CINAHL, Applied Social Sciences Index and Abstracts, International Bibliography of the Social Sciences, PsycINFO, Web of Science, ERIC and citation search. Quantitative or mixed-methods studies that reported the correlates of mobile device use or PSU on children aged 6 or younger. Studies were excluded if they focused on children with critical illnesses or developmental disorders, combined mobile device use with other forms of screen time (such as television), assessed the health impacts of device use, were conducted in laboratories or during the COVID-19 pandemic, or were not published in peer-reviewed journals. Screening, data extraction and quality assessment were performed independently by two reviewers. The correlates were broadly classified across three levels of the socioecological model. Random-effects meta-analyses were performed using the Fisher transformation of the correlation coefficient.

**Results:**

A total of 39 studies with 32 927 individuals from 17 countries were included. 115 correlates were identified: 43 at the individual level, 68 at the interpersonal level and 4 at the environmental level. No correlates revealed a significant effect for PSU. However, there was a significant positive association between mobile device use and: (1) Age (tablet *r*=0.211 (95% CI 0.006 to 0.416); smartphone *r*=0.107 (95% CI 0.035 to 0.179); combined *r=*0.182 (95% CI 0.063 to 0.302)); (2) sex (boys) (smartphone *r*=0.051 (95% CI 0.010 to 0.101)); (3) parental device use (tablet *r*=0.417 (95% CI 0.290 to 0.544); smartphone *r*=0.237 (95% CI 0.134 to 0.340)) and (4) parental stress (smartphone *r*=0.168 (95% CI 0.065 to 0.271)).

**Conclusion:**

Future studies addressing mobile device use in young children should consider parental device usage and provide support to reduce parental stress.

**PROSPERO registration number:**

CRD42024543727.

**WHAT IS ALREADY KNOWN ON THIS TOPIC**

- Mobile screen devices are prevalent in young children’s lives. Systematic reviews have shown that increased use of mobile devices is associated with adverse health outcomes, such as myopia, poor sleep quality and behavioural issues. It is important to understand the factors (eg, individual, family, childcare and neighbourhood) that influence mobile device usage among children, in order to promote healthier behaviours.

**WHAT THIS STUDY ADDS**

- This systematic review and meta-analysis reported a significant association between parental mobile device use and the time children under 6 spend on their devices. It also demonstrates that a child’s age, sex and parental stress significantly influence device usage.

**HOW THIS STUDY MIGHT AFFECT RESEARCH, PRACTICE OR POLICY**

- This systematic review emphasises the need to address parents’ use of mobile devices as a strategy for reducing children’s device usage.
- Future studies and interventions should examine and prioritise parental device usage and develop strategies to reduce parental stress while enhancing parenting skills to promote healthier mobile device usage among young children.

## Introduction

 Mobile screen devices have become an integral part of young children’s lives, with recent data indicating that 90% of children aged 3–4 years go online via tablet (75%), mobile phone (41%), handheld games (11%), laptop (8%) and desktop (4%).^1^ The portability and size of these devices, combined with the interactive educational and entertainment opportunities they offer, make them appealing to both parents and children.^2 3^

Numerous systematic reviews have gathered evidence regarding the effects of mobile device usage on health outcomes for young children. These reviews have reported that increased mobile device use is associated with a higher risk of myopia,^4^ poor sleep quality,^5^ as well as behavioural issues, low academic performance and socioemotional challenges.^6^ However, mobile device use may also provide benefits, particularly in learning environments, as it can effectively enhance literacy, numeracy, manual dexterity and visuo-spatial working memory.^7^ Likewise, a meta-analysis revealed a small positive association of adult-child co-use on children’s learning from digital media compared with children’s solitary digital media use.^8^ In 2019, the WHO issued guidelines regarding screen time for children. The guidelines stated that children under 2 should have no exposure to screens, while those aged 3–4 should limit screen time to no more than 1 hour per day.^9^ However, a meta-analysis that included 44 studies across various countries—20 from Europe, 12 from Australia, 8 from North America and 4 from Asia—revealed that only 24.7% of children younger than 2 years and 35.6% of children aged 3–5 meet the WHO guidelines.^10^

To effectively change behaviour, it is essential to first understand the underlying factors that drive and influence behaviour.^11 12^ This entails identifying the individual, sociocultural and environmental factors that are most likely to affect mobile device usage.^13^

An extensive systematic review in 2010 (n=71 studies) identified several strong correlates of screen viewing among young children, including demographic factors (eg, child age, ethnicity); social-cultural influences (eg, father’s educational level, family television (TV) viewing, parental rules) and environmental aspects (eg, neighbourhood safety).^14^ However, this review was published over a decade ago and focused only on TV viewing, computer use and video games. In recent years, the percentage of children 4–15 years old watching TV has significantly declined, from 76% in 2019 to 53% in 2024.^15^ Meanwhile, the use of interactive electronic devices has surged, with 69% of children aged 3–5 years in the UK using tablets and 34% using mobile phones.^1^ The 2025 Common Sense Census report highlights a significant disparity in screen time among children in the USA based on household income. Children from lower-income households spend an average of 3 hours and 48 min per day on screens, approximately twice the 1 hour and 52 min observed for children from higher-income households.^16^ The evidence across the world regarding screen use by young children varied, with the majority of studies originating from high-income countries, particularly Europe, Australia and North America.^17^

More recently, a systematic review conducted by Paudel *et al*^18^ examined the correlates of mobile screen media use among children aged 8 years and younger. The findings indicated that higher mobile screen media use was positively correlated with the child’s age, their skills in using these devices, access to mobile devices at home and parental usage of mobile screen devices. Additionally, a three-level meta-analysis revealed that the provision of social support, especially from family, shows a significant negative correlation with problematic mobile phone use among children and adolescents.^19^ Problematic smartphone use (PSU) is characterised by uncontrolled and excessive usage that negatively impacts an individual’s life.^20^ For instance, a study involving preschool children in South Korea found that 17% (236 out of 1378 children) met the criteria for PSU.^21^ A systematic literature review examining problematic media use (PMU)—which includes devices such as computers, video games, smartphones, tablets and TVs—found that PMU can lead to negative effects on children’s development (ie, emotional intelligence), as well as their well-being (ie, problematic behaviours, sleep problems and depressive symptoms).^22^ As children age and transition into their teenage years, the prevalence of mobile device usage and PSU tends to rise.^23 24^ This underscores the importance of understanding the factors that influence this behaviour during early developmental stages when such habits are still forming. The aim of this systematic review and meta-analysis is to identify and quantify the correlates of mobile screen media use, including duration of mobile device use (specifically tablets and smartphones) and PSU, among children aged 0–6 years. We employed a socioecological model^25^ to capture the multilevel factors that correlate with and influence children’s mobile device use, considering how networks of people and structures surrounding the child (eg, family, childcare, neighbourhood) affect this behaviour. The socioecological model acknowledges that individuals exist within a larger, interactive social system, which significantly influences behavioural outcomes.^26^ By using this framework, we can identify specific factors related to mobile phone usage and PSU at different levels, which may help guide future interventions.^27^

This systematic review aims to update the previous review by Paudel *et al*^18^ which was based on a database search conducted in March 2017. This updated review will include additional databases (eg, ERIC), incorporate studies from childcare settings that were previously excluded and conduct a meta-analysis.

## Materials and methods

The study was conducted in accordance with the Preferred Reporting Items for Systematic Reviews and Meta-analyses (PRISMA) guidelines^28^ (online supplemental file 1) and performed according to an a priori protocol registered in PROSPERO (CRD42024543727) (online supplemental file 2).

### Search strategy and selection criteria

Studies were included if they focused on children aged 6 years or younger, as this is the approximate age at which children begin compulsory education. Additionally, studies that included data within a broader age range, which separately provided the 0–6 years data, were also considered. We included quantitative (either observational or intervention) as well as mixed-methods studies from which we extracted the quantitative components. The study had to report the correlates or determinants of mobile device use duration, measured in minutes or hours per day, for smartphones and tablets, either separately or together. We also included studies that reported PSU.

We excluded studies involving children with serious illnesses or developmental disorders. We did not include studies that combined mobile device use with other screens, unless they provided separate data for mobile devices. We also omitted studies examining the impact of device use on health, as well as those conducted in labs or during the COVID-19 pandemic without prepandemic data. Finally, we excluded conference abstracts and non-peer-reviewed studies.

A systematic literature search was conducted by the authors MC and EP in June 2024 and updated in March 2026 on the following databases: MEDLINE, Scopus, Embase, CINAHL, Applied Social Sciences Index and Abstracts, International Bibliography of the Social Sciences, PsycINFO, Web of Science and ERIC.

The search strategy used specific terms related to the population (children aged 0–6 years), exposure (mobile screen devices) and correlates (not limited to a particular correlate, use synonyms of the correlate word). These concepts were searched for using subject headings (where possible) and free-text terms, which were then combined using Boolean operators. The searches were restricted to the English language, with no date limit imposed. The search terms and complete search strategies are listed in online supplemental file 3. The reference lists of retrieved papers and recent reviews were manually searched to find additional studies.

All retrieved records were imported into Covidence (www.covidence.org). Two rounds of screening (ie, titles and abstracts and full-text) were conducted independently by two researchers from the team (LBdA, JS, DJ, CM, AH). If any disagreements arose during the process, they were resolved through discussion or consultation with the main author (LBdA) until a consensus was reached.

### Data extraction and quality assessment

A bespoke data extraction template was piloted across reviewers. The following information was extracted: publication details (eg, author, title, year of publication), population characteristics (eg, age, sex, ethnicity), location (country), study design, sample size, type and method of assessing mobile screen device use (duration and/or content), outcome measures, results regarding the association between mobile screen device use and correlates (eg, correlation coefficients, regression coefficients, risk or OR). For studies with a longitudinal design, the final data point was extracted for analysis. Data were extracted by one researcher (LBdA, JS, DJ, CM, AH) and verified by the first author (LBdA).

The quality of eligible studies was systematically assessed against the Critical Appraisal Skills Programme checklists for cross-sectional or cohort studies. Quality assessment was conducted by one of the authors (DJ, JS, CM, AH) and the first author (LBdA), and disagreements were resolved by consensus.

### Data analysis

Both narrative synthesis and meta-analysis were conducted to explore the correlates of mobile device use in young children.

#### Narrative synthesis

A narrative synthesis was undertaken to integrate the findings guided by Synthesis Without Meta-Analysis.^29^ Correlates of mobile device use were broadly classified across three levels of the socioecological model^25^: (1) individual (child); (2) interpersonal (parent/carer); and (3) environment (community, organisational and policy).

To assess the strength of the association between mobile device use (smartphones and tablets, both separately and together) or PSU with each potential correlate, we examined the percentage of studies reporting an association in a specific direction. If three or more studies had tested an association, and 0–33% reported significant associations in a positive or negative direction, the result was categorised as no association and marked as ‘0’. If 34–59% reported significant associations in a consistent direction, the result was categorised as inconsistent, marked as ‘?’. If 60–100% reported a significant association in a consistent direction, the result was coded as positive ‘+’ or negative ‘-’.^30^ For correlates reported in three or fewer studies, they were still listed, but synthesis results were regarded as insufficient to derive a meaningful association. The overall associations across the studies were categorised based on the consistency of the findings. If more than 60% of the results indicated positive, negative or null, the most consistent would be reported. (eg, n=5 (40%+^study 1, study 2^; 60% 0 ^study 3, study 4, study 5^); overall 0 (null association)). If the proportion of consistent findings across studies was between 33% and 60%, the overall association would be classified as inconsistent (eg, n=4 (50%+^study 6, study 7^, 50% − ^study 8, study 9^);? (inconsistent)). Conversely, if none of the studies showed an association above 33%, the overall association would be considered null (eg, n=3 (33%+^study 10^, 33% − ^study 11^, 33% 0 ^study 12^); 0 (null association)).

#### Meta-analysis

Correlational meta-analyses were conducted on all correlates associated with the duration of mobile device use or PSU. The studies included in the meta-analyses reported their results using various statistical measures such as covariances, correlation coefficients, standardised regression statistics, the χ² statistic, ORs or t-statistics. The equations used in the derivation of effect statistics can be found in online supplemental file 4.

Meta-analyses were performed for the duration of tablet use and smartphone use, both separately and combined. When studies reported screen time for tablets and smartphones separately, these measures were converted into a combined measure of tablet/smartphone use using an expression that relates a correlate to the sum of the two other measures:


$$r\left(A,B+C\right)=\frac{r\left(A,B\right)\cdot \sigma_{B}\;+\;r\left(A,C\right)\cdot \sigma_{C}}{\sqrt{\sigma_{B}^{2}+\sigma_{C}^{2}\;+\;2\cdot r\left(B,C\right)\cdot \sigma_{B}\cdot \sigma_{C}}}$$

where

A=correlate of interest.

B=variable representing the duration of tablet use by the child.

C=variable representing the duration of smartphone use by the child.

Where a value for the correlation coefficient was not provided, an estimate was used based on a meta-analysis of the correlation between these measures across all included studies that provided this information.

Meta-analyses were conducted subject to a minimum of three relevant studies identified for inclusion. In instances where studies reported separate parent-level correlates for mothers and fathers, only the maternal data were included in the analysis.

Correlation coefficients from primary study data were transformed before being entered into a meta-analysis using Fisher’s Z transformation (online supplemental file 4). Random effects models were conducted on grouped data, reflecting identified clinical and methodological heterogeneity between studies.

### Role of the funding source

The funder of the study had no role in study design, data collection, data analysis, data interpretation or writing of the report.

## Results

**Figure 1:**
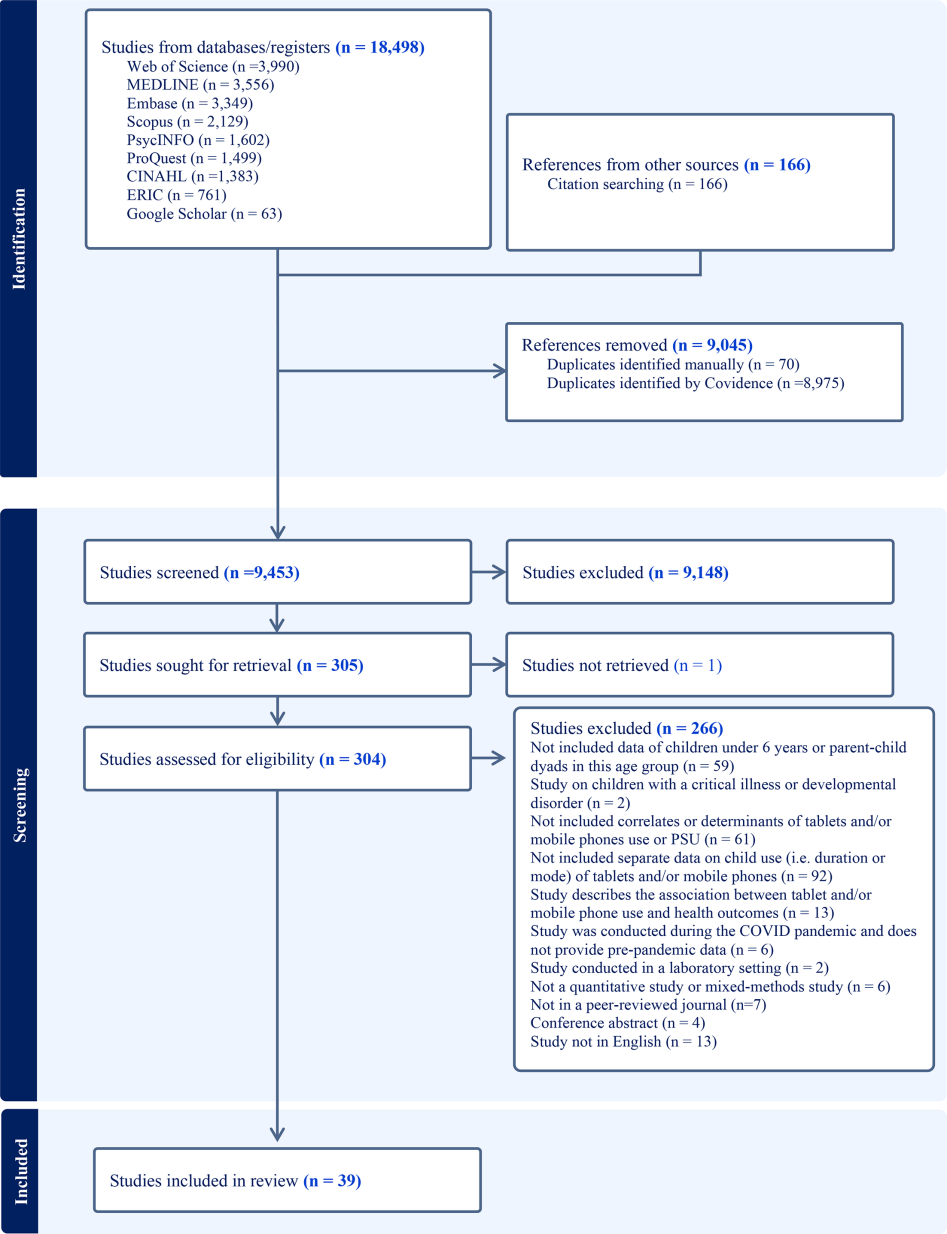
Selection of studies for inclusion in the systematic review. PSU, problematic smartphone use.

The search yielded a total of 18 498results, of which 304 were read in full and 39 studies met the inclusion criteria (figure 1). A total of 32 927 individuals were included across the studies. The sample size ranged from n=33^31^ to n=4907.^32^

### Summary of study characteristics

Detailed information about the included study is provided in online supplemental file 5. The included studies were published between 2013 and 2026. The country which had the largest number of studies was South Korea (seven studies),^2133^,^38^ followed by the USA (six studies),^39^,^44^ and Turkey^45^,^48^ (four studies), Portugal^45^,^4749^ (three studies), Argentina,^31 52^ China,^32 53^ Malaysia^54 55^ and Thailand^56 57^ (two studies each), and one study published in each of these countries^52^: Australia,^58^ Bangladesh,^32 59^ Finland,^60^ Jordan,^61^ Netherlands,^62^ Philippines,^63^ Singapore,^64^ Spain^65^ and Sri Lanka.^66^ Two studies were conducted on the population across two countries, China and Australia^67^ and Australia and the USA.^68^

35 studies used cross-sectional analysis,^2131^,^37 40 41 43^ while 4 employed a longitudinal design.^38 39 42 53^ One study conducted a cross-sectional analysis using data from a longitudinal survey.^40^ Six studies focused exclusively on children younger than 3 years old.^31 35 39 42 43 52^ We extracted parental education as a proxy for socio-economic status. Out of the 24 studies that provided data on parental education,^3336 37 40 42^,^44 46 47 50 51 53^ 3 included participants in which the majority did not possess a college or university degree.^55 56 66^

The duration of mobile phone and tablet use was reported in 19 studies.^2131 32 34 41 44 49 51^,^53 56 57 60^ The average time spent on these devices varied, ranging from 19.88 (SD 40.95) min/day for smartphones^65^ to 144 (106.95) min/day for tablets and smartphones.^63^ In 12 studies,^35^,^3840 42 43 45 47 48 59 66^ data were presented as categorical (eg, usage <30 min; 31 min–1 hour; 1–2 hour; 2–3 hour; and 3–4 hour). Eight studies did not report on the duration of mobile device use.^33 39 46 50 54 55 58 64^ Only 13 of the included studies provided information on the content children were watching,^2131 37 45 54 56^,^59 63 67^ and this was reported in different ways. Some reported the percentage of time spent on each type of content,^31 57 63^ while others listed the content.^37^

Eight studies examined the correlates of PSU,^21 34 35 38 39 54 59 64^ while 30 explored correlates of mobile device time.^31^,^3336 37 39^ One study looked at the correlates of both screen time and PSU.^39^ Among the studies that explored the correlates with mobile device use, 13 studies explored the association with combined smartphone and tablet use,^3240 44 47 48 51 53 55^,^57 60 62 63^ while 11 explored the separate associations with tablet and smartphone.^21 31 41 42 46 49 50 52 58 67 68^ Six studies explored the association with smartphones only,^33 37 45 61 65 66^ and two studies explored the association with tablets solely.^39 43^

Different questionnaires were employed to measure PSU.^3869 71^,^73^ Some studies^54^ adapted their questionnaire^72^ to their population’s cultural background, while others^64^ modified the questionnaire for a younger population.^73^ Additionally, Abdullah *et al*^59^ created a new questionnaire based on various existing questionnaires reported in the literature.^74^,^76^

Most studies developed customised questionnaires to measure mobile device time.^31^,^3336 37 39 42 43 45 46 52 56 60^ Some studies^47 57 68^ used validated or internally validated questionnaires,^38 44 77 78^ while others^4149^,^51 55 58^ adapted questionnaires from other surveys^53 70 79 80^ or from media organisations.^81^ One study used a passive sensing app for Android devices and screenshots for iOS devices.^82^

Across all the included studies, this systematic review identified a total of 115 correlates. Among these, 99 correlates pertained specifically to mobile device use duration, while 31 were connected to PSU, with 13 correlates investigated on both duration and PSU. 45 correlates were investigated at the individual level, 68 at the interpersonal level and 4 at the environmental level. Correlates were synthesised using a meta-analysis and a narrative synthesis.

### Quality assessment

Only 3^21 44 49^ of the 39 studies included met all the quality scores assessed (online supplemental file 6). Among the cross-sectional studies (n=35), most studies addressed a clear, focused issue and presented a clear statement of the findings (89% of included studies). However, very few clearly stated whether recruitment was conducted in an acceptable manner (37%), provided evidence of having enough participants to minimise the influence of chance (31%) or demonstrated that the study results could be generalised (29%).

All four longitudinal studies presented a clear and focused issue, considered confounding factors, communicated their results effectively and compared their findings with existing evidence and implications for practice. However, none of the included studies reported on the outcomes of those participants who left at follow-up.

### Data synthesis

Data synthesis of PSU and duration of mobile device use was conducted using two methods: (1) narrative synthesis; (2) meta-analysis.

### Narrative synthesis

#### Problematic smartphone use

Four correlates of PSU were identified at the individual level, and 25 correlates at the interpersonal level. Among these, five correlates were found in three or more studies and are reported in table 1. Three out of four studies that investigated the association between PSU and parent use of mobile devices reported a positive association.

**Table 1:** Summary of individual and interpersonal correlates of problematic smartphone use

Correlates	Problematic smartphone use Number of studies (percentage, direction of association, study citation); overall association
Individual	
Age	n=6 (40% + ^36 54^; 65% 0^38 59 64^); 0
Sex (boys)	n=4 (25% + ^59^; 25% -^64^; 50% 0^21 38^); ?
Interpersonal	
Income	n=5 (20% + ^54^, 20% -^21^; 60% 0^38 39 59^); ?
Parents educational level	Mother education: n=2 (50% + ^59^; 50% –^64^); +;
	Father education: n=1 (100% + ^59^); +;
	Parent education:
	n=1 (100% 0^54^); #;
	Overall: n=3 (33% + ^59^, 33% –^64^, 33% 0^54^); ?
Parental device use	Mother’s use: n=2 (50% + ^59^, 50% –^34^); #;
	Father’s use: n=1 (100 % + ^59^); #;
	Parent’s use: n=1 (100% + ^35^); #
	Overall: n=3 (75% + ^34 35 59^, 25% –^34^); +

Note 1: Association codes: 0, no association; ?, inconsistent; –, negative; +, positive; #, insufficient data (<3 studies) to derive an association.

Note 2. If the same study provides correlations with maternal, paternal and parental, the calculation of overall association prioritises the maternal correlation first, followed by the paternal and then the parental.

Online supplemental file 7 reports an additional 26 correlates that were investigated in fewer than three studies.

#### Duration of mobile device use

11 correlates related to the duration of mobile device use were identified across three or more studies. Child TV duration and parental use of mobile devices were positively associated with child mobile device use (table 2 and online supplemental file 8). An additional 88 correlates were explored in three or fewer studies (online supplemental file 9).

**Table 2:** Summary of individual and interpersonal correlates with the duration of mobile device use

Correlates	Overall association
Individual	
Age	n=11 (54% + ^41 43 44 51 55 63^, – 9% –^65^; 37% 0^36 37 56 67^); ?
Sex (boys)	n=8 (14%+ ^66^, 86% 0^32 36 37 45 55 67^); 0
Child TV use duration	n=3 (66.6%+ ^39 51^; 33.3%0^52^); +
Interpersonal	
Family characteristics and structure	
Parental age (older)	n=5 (60% 0^43 53 55^; 20% + ^45^; 20% –^58^); ?
Parents educational level	n=12 (33% –^32 45 51 55^; 17% + ^52 66^; 50% 0^36 43 53 58 60 67^); ?
Employment	n=6 (100% 0^33 36 45 52 55 66^); 0
Income	n=11 (36%–^39 45 49 55^; 64% 0^32 36 43 58 60 66 67^); 0
Presence of siblings	n=5 (20% + ^55^; 80% 0^32 43 53 67^); 0
Family rules and behaviours	
Parental device use	n=9 (100%+ 4143 44 56,58 61 62 68); +
Parental stress	n=3 (100% 0^33 42 58^); 0
Parental depression	n=4 (100% 0^33 36 53 58^); 0

Data reported across all devices, including number of studies (percentage, direction of association, study citation); overall association.

Note 1: Association codes: 0, no association; ?, inconsistent; –, negative; +, positive; #, insufficient data (<3 studies) to derive an association.

Note 2: In the overall analysis, when studies reported data for both combined and separate devices, the combined data were prioritised. If data were provided only separately for tablets and smartphones, tablets were given priority in calculating the overall score.

Note 3: If the same study provides correlations with maternal, paternal, parental or relative, the calculation of overall association prioritises the maternal correlation first, followed by the paternal, the parental and then the relative.

Note 4: Data per device is reported in online supplemental file 8.

TV, television.

### Meta-analysis

The meta-analysis was conducted in 7 studies, which assessed PSU^21 34 35 39 54 59 64^ and 22 studies that measured the duration of mobile device use.^3233 37 39 41 43^,^45 49^

#### Problematic smartphone use

**Figure 2:**
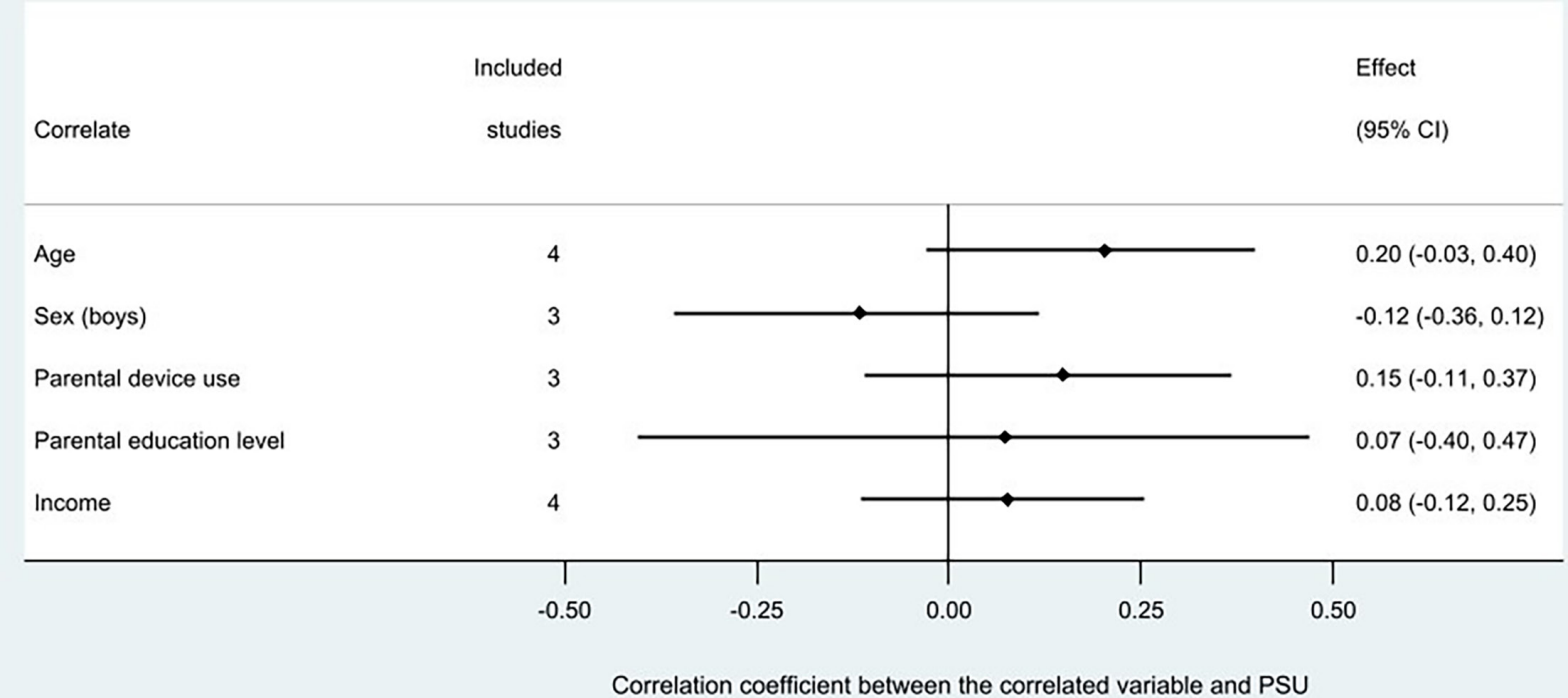
Correlates of PSU. PSU, problematic smartphone use.

No statistical significance was found among the correlates with PSU, including age, sex, parental mobile device use and family income. A significant amount of heterogeneity was observed across all correlates, exceeding 98% (figure 2 and online supplemental file 10).

#### Duration of mobile device use

**Figure 3:**
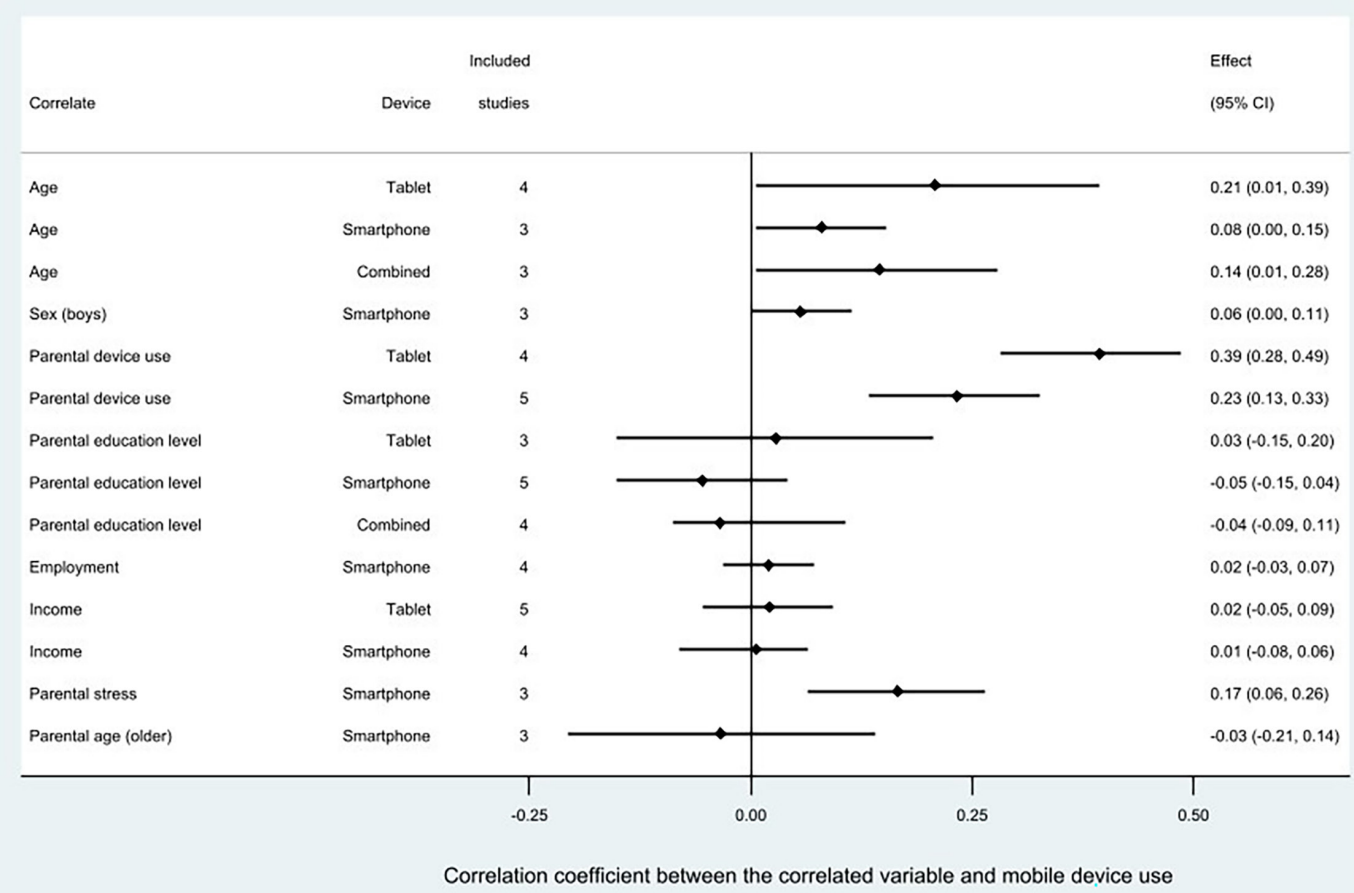
Correlates of mobile device use.

A series of meta-analyses examined the association between various correlates and the duration of tablet, smartphone and combined device use (figure 3 and online supplemental file 8). A significant correlation was revealed between: (1) age with tablet, smartphone and combined use; (2) sex (boys) with smartphone use; (3) parental mobile device use with child tablet and smartphone use; and (4) parental stress levels with child smartphone use.

The most substantial association was observed between parental mobile device use and children’s tablet usage (r=0.417) and smartphone use (r=0.237). There was a wide range of heterogeneity observed, from 0% (parental employment and smartphone use) to 98% (child age and tablet use).

## Discussion

This systematic review and meta-analysis identified a significant association between parental mobile device usage and the duration of mobile device use in children under 6 years old. The analysis also revealed that factors such as child age, sex and parental stress significantly correlate with the duration of mobile device use among young children. In terms of PSU, although no statistically significant differences were found in any of the meta-analyses conducted, the narrative synthesis indicated that most studies (three out of four) that examined the relationship between PSU and parental device use reported a positive association.

This systematic review included 39 studies and identified 115 correlates with the duration of mobile device use or PSU in young children. A previous systematic review by Paudel *et al*^18^ included 13 studies and mapped 36 correlates. Given the rising trend of mobile phone usage among young children in recent years, it is not surprising that more studies are now focusing on this topic and examining additional correlates. Their narrative review concluded that both age and parental use of mobile devices were associated with mobile device usage in young children, a finding that our meta-analysis confirmed. In our systematic review, we found that 25 of the 39 included studies were conducted in developed countries.^2132^,^43 48^ Nevertheless, there is also a growing research interest from developing countries, as evidenced by the inclusion of 14 studies from these countries.^3145^,^48 52 54^

Similarly, a meta-analysis which included studies in older children and adolescents aged 7–19 years old reported a negative association between social support and PSU.^19^ Although social support was not identified as a correlate in our systematic review, possibly due to the target age group lacking the capacity to perceive social support,^83^ our meta-analysis did show a positive association between parental stress and the duration of mobile device use in children. Parenting stress in the studies included in this review were measured with scales which assessed the stress within the parent–child relationship.^84^ It appears from the results of this association that when parents experience stress in their relationship with their child, they may resort to giving the child an electronic device as a means of alleviating this stress.^42^ However, it is important to recognise that this relationship may be bidirectional, and reliance on devices for coping can lead to parental withdrawal from interactions, potentially exacerbating problematic behaviours in children.^85^ Recently, the American Psychological Association has proposed various strategies for parents to effectively manage stress and burnout.^86^

It is also important to note that while our systematic review reported the duration of mobile device use and PSU separately, some studies have indicated that PSU is related to both the frequency and duration of mobile device usage among young children.^21^ Therefore, findings regarding PSU can be cautiously compared with the duration of mobile device use.

Age emerged as another significant correlate associated with mobile device use in young children. According to Ofcom,^87^ the percentage of children who own a mobile phone increases almost linearly from the age of 3–12 years. By the time children are between 12 and 15 years old, 96% have their own mobile device. Our meta-analysis further revealed that boys are more likely than girls to use mobile devices. This finding contradicts previous systematic reviews that found no association between sex and smartphone use in children or adolescents.^18 88^ It remains unclear why boys at this age might use devices more than girls. National surveys indicate that boys typically engage with video games at an early age.^1^ However, as children grow older, there is no difference in mobile device use between boys and girls. While boys tend to continue using devices primarily for gaming, girls are more likely to use them for social interaction.^89^

We synthesised the evidence for each device type (eg, tablet and smartphone) or combined data, following the reporting in the original studies. However, our meta-analysis did not find differences based on the devices used. Nevertheless, some studies that examined associations per device reported varied results. For example, Gago-Galvano *et al*^52^ observed a positive correlation between the use of tablets by children and mothers’ educational level, whereas no correlation was identified between the use of smartphones by children and maternal education. These differences in correlates based on device type are likely related to whether a child has one or both devices and their preferences regarding device type, rather than intrinsic differences in correlates by device type; however, future studies could further explore this area.

A key strength of this study is its comprehensive mapping of all covariates reported in the literature regarding mobile device use or PSU in young children. This review identified 115 correlates (99 duration of mobile device use, 31 PSU and 13 in both). However, only five correlates for PSU and eight for mobile device use were reported in at least three studies and had the necessary statistics for inclusion in the meta-analysis. Nevertheless, the mapping of these correlates not only provides us with an understanding of the variables being investigated in the literature but also offers insight into variables that appear as potential correlates but require further evidence. For instance, parent efficacy was found to be negatively correlated with mobile device use in both studies that examined this variable^32 37^ (online supplemental file 8). Therefore, further research is needed to explore this relationship in more detail. Another notable strength of this systematic review is its comprehensive search across a wide range of databases, and as the first to employ a meta-analysis.

### Strengths and limitations

To the authors’ knowledge, this study represents the first known meta-analysis examining the correlates of problematic smartphone and mobile device use in young children. An extensive literature search was conducted, encompassing nine databases without any restriction on publication dates.

Nonetheless, the study presents several limitations. We restricted the search to English-language studies, which may have led to the omission of critical data and limited the generalisability of our findings. Likewise, we were constrained from further analysing the data by country socio-economic level due to the limited number of studies from developing countries.

Within the literature included in the systematic review, we observed a high level of statistical heterogeneity observed among certain correlates (online supplemental file 10). The studies included in this systematic review reported their analyses either separately by device or in combination, and used different measurement tools for PSU and the duration of mobile device use. Most studies reported mobile device use duration through customised questionnaires, while only one study^40^ used a passive sensing app for direct measurement. Similarly, the correlates reported in the included studies used different tools, for example, to assess income, parent stress or parental depression. This variability might have contributed to the heterogeneity reported in the meta-analysis.

Another limitation is the lack of longitudinal studies, with only four included in this review. Furthermore, the quality assessment revealed that cross-sectional studies lacked information on their recruitment strategies and power calculations. In contrast, longitudinal studies did not provide adequate details on participant characteristics at follow-up.

## Conclusion

This review presents compelling evidence from both a meta-analysis and a narrative synthesis, showing that parents’ use of mobile devices is associated with children’s device use. The literature suggested that parent smartphone use affects parental sensitivity and responsiveness to young children,^90^ emphasising the need to address this behaviour. Other correlates identified include the child’s age and sex, as well as parental stress levels.

We recommend that in the future primary intervention studies targeting mobile device use in children should focus on parental usage and provide support to reduce parental stress and enhance parenting efficacy. Moreover, there is a need for more longitudinal studies and better measurement of screen behaviours, potentially through the utilisation of passive sensing apps.

## Supplementary material

10.1136/bmjph-2025-004305 online supplemental file 1

10.1136/bmjph-2025-004305 online supplemental file 2

10.1136/bmjph-2025-004305 online supplemental file 3

10.1136/bmjph-2025-004305 online supplemental file 4

10.1136/bmjph-2025-004305 online supplemental file 5

10.1136/bmjph-2025-004305 online supplemental file 6

10.1136/bmjph-2025-004305 online supplemental file 7

10.1136/bmjph-2025-004305 online supplemental file 8

10.1136/bmjph-2025-004305 online supplemental file 9

10.1136/bmjph-2025-004305 online supplemental file 10

## Data Availability

Data sharing not applicable as no datasets generated and/or analysed for this study.
