# Supplementary material for: Correlates of mobile device use in young children: a systematic review and meta-analysis
Source: BMJ Public Health. 2026 Jun 17;4(2):e004305. doi: 10.1136/bmjph-2025-004305 (PMC13289221; doi:10.1136/bmjph-2025-004305)
Supplement: online supplemental file 2 [file bmjph-4-2-s002.pdf]

NIHR | National Institute for Health and Care Research

PROSPERO
International prospective register of systematic reviews

Correlates and determinants of mobile screen device use in young children aged 0-6 years: a systematic review and meta-analysis

Liane B. Azevedo, Elizabeth Goyder, John Stephenson, Daniel Jones, Mark Clowes, Colette Marr

Citation
Liane B. Azevedo, Elizabeth Goyder, John Stephenson, Daniel Jones, Mark Clowes, Colette Marr. Correlates and determinants of mobile screen device use in young children aged 0-6 years: a systematic review and meta-analysis. PROSPERO 2024 Available from https://www.crd.york.ac.uk/PROSPERO/view/CRD42024543727

REVIEW TITLE AND BASIC DETAILS

Review title
Correlates and determinants of mobile screen device use in young children aged 0-6 years: a systematic review and meta-analysis

Review objectives
What are the socio-ecological factors associated with interactive electronic device use in young children (6 years and under)?

Keywords
Correlation, Early years, Interactive electronic devices, Socio-ecological model, Young children

SEARCHING AND SCREENING

Searches
A literature search will be carried out using the following databases: MEDLINE, Scopus, Embase, CINAHL, ProQuest, PsycINFO, Web of Science, ERIC, Applied Social Sciences Index and Abstracts and Sociological Abstracts with no date restriction. We will only include studies published in English. No date restriction will be applied.
We will have two sets of search terms, the first related to population (i.e., child*; kid*; Preschool* ; Pre- school* ; Nurser*; Toddler*; Kindergarten*; Early years; Early childhood; Infant; Childcare) and the second to mobile screen device (i.e. smartphone*; mobile phone*, cell phone*, tablet*, iPad*, handheld media, handheld computer*; interactive electronic devices). We

will not include search terms for resultants of mobile screen device use as we want to be as open as possible to potential resultants.

Study design

We will include peer-reviewed quantitative studies (observational and intervention studies), which reported the correlates/determinants of mobile screen device use. Qualitative studies, systematic reviews, non-human studies and conference abstracts will be excluded.

ELIGIBILITY CRITERIA

Condition or domain being studied

Mobile screen devices are part of young children's lives, with recent data reporting that 90% of children aged 3-4 years go online (Ofcom, 2022). A systematic review (Paudel et al., 2017) revealed that higher mobile screen media use was positively associated with the child's age, their skills in using these devices, access to mobile devices at home, and those with parents with high use of mobile screen devices. Understanding the correlates and determinants of mobile screen devices could inform the multi-level factors that influence children's use and guide future design and content of interventions.

In this systematic review, we will focus on the age range of 0 to 6 years, which has been defined as the early years age range. We will use the socio-ecological model (Ryan 2001) to capture the multi-level factors that correlate with and/or influence children’s use of mobile screen devices and how networks of people and structures surrounding the child (e.g., family, childcare, neighbourhood) would influence mobile screen use.

References:

Ofcom UK. 2022. Adults’ media use and attitudes report.

Paudel et al. 2017. Correlates of mobile screen media use among children aged 0–8: a systematic review. BMJ open. 2017

Ryan 2001. Bronfenbrenner’s ecological systems theory;9:2012.

Population

We will include studies of children aged 6 years or less or studies which included parent-child dyads in this age group. We will also include studies that present data on a wider age range but report data on the age category that fits our inclusion criteria (0 to 6 years).

We will exclude studies which only present data on children suffering from a critical illness or present a developmental disorder (e.g., allergy, asthma, cerebral palsy, cystic fibrosis, autism, ADHD).

Intervention(s) or exposure(s)

We will include studies that quantify the association between correlates (cross-sectional association) or determinants (prospective association) of hand-held mobile device use (i.e., duration of use or content watched) in young children (0 to 6 years). By hand-held mobile use, we mean mobile touchscreen devices, tablets, and mobile phones (e.g. smartphones). Studies which reported the use of mobile screen devices with non-interactive or non-hand-held electronic devices, for example, television, laptop or computer, will be excluded.

Comparator(s) or control(s)

Not applicable

Context
We will include studies conducted in any country and performed in any setting, including childcare, schools and homes. However, we will exclude studies conducted in laboratory settings.

OUTCOMES TO BE ANALYSED

Main outcomes
The systematic review and meta-analysis will include studies that explore the individual (e.g., age, gender, ethnicity), interpersonal (e.g., maternal education; parenting style), environmental (e.g. neighbourhood safety; neighbourhood greenery), and policy (e.g., childcare or school policies) correlates or determinants of mobile screen device use.
The use of mobile screen devices may encompass duration (e.g., minutes/hours per day/week) and/or content (e.g., game apps and streaming videos).

Measures of effect
Correlation coefficients, regression coefficients, risk ratio or odds ratio.

Additional outcomes
None

Measures of effect
Not applicable

DATA COLLECTION PROCESS

Data extraction (selection and coding)
Studies identified through database search will be exported to Covidence (www.covidence.org), where duplicates will be automatically removed. This will be followed by two rounds of screening, including the first title/abstract screening and full-text screening. Two researchers will complete the screening independently. Eligibility will be assessed based on the inclusion and exclusion criteria. Disagreement will be resolved by discussion or consultation with another researcher until a consensus is reached. We will use the PRISMA flowchart to record the selection process.
A bespoke data extraction template will be piloted across reviewers. The following information will be extracted: publication details (e.g., author, title, year of publication), population characteristics (e.g., age, gender, ethnicity), location (country and study setting), study design, sample size, type and method of assessing mobile screen device use (duration and/or content), outcome measures (correlates or determinants), statistical information (e.g., analysis, controlling variables), results regarding the association between mobile screen device use and correlates or determinants (e.g., correlation coefficients, regression coefficients, risk or odds ratio). We will generally include unadjusted regression coefficients and bivariate correlation coefficients in meta-analyses to minimise heterogeneity arising from varying levels of control. Data will be extracted independently by two reviewers.
In case of missing data, we will search for other related publications from the same study and/or supplementary materials. If data is not available, respective authors will be contacted. If data cannot be retrieved, the study might be included in the narrative synthesis but will be excluded from the meta-analysis.

Risk of bias (quality) assessment

The quality of eligible studies will be systematically assessed against the Critical Appraisal Skills Programme (CASP) checklists appropriate for the study design to appraise the trustworthiness, relevance, and results of eligible studies. Two reviewers will perform quality assessment independently, and a third reviewer will resolve any disagreements.

We will also assess the quality of the total body of evidence using the Grading of Recommendations Assessment, Development and Evaluation (GRADE) framework. The quality of evidence will be categorised into "high," "moderate," "low," and "very low." Two reviewers will complete this process independently, and a third reviewer will resolve disagreements, if necessary.

PLANNED DATA SYNTHESIS

Strategy for data synthesis

If possible, we will conduct meta-analyses on all identified outcomes, subject to the provision of suitable data, illustrating results via forest plots. We will consider both random- and fixed-effects meta-analyses for each outcome based on clinical and methodological heterogeneity identified a priori. We will not make post hoc decisions on the type of analysis (i.e. random effects or fixed effects) based on heterogeneity tests. For random effects meta-analyses, heterogeneity statistics will also be reported, including Cochran's Q test for heterogeneity, the I^2 statistic (proportion of variation across studies ascribed to heterogeneity) and the τ2 statistic (an estimate of between study variance), from which prediction intervals may be calculated.

Sensitivity analyses will be conducted for all outcomes to assess the robustness of the derived estimates and illustrated on influence plots. Subject to 10 or more studies being identified for inclusion in a particular meta-analysis, funnel plots will be constructed to assess small-study effects.

Regardless of whether meta-analyses are conducted, a narrative synthesis will be undertaken to integrate the findings guided by Synthesis Without Meta-Analysis (SWiM) (Campbell et al. 2020). Correlates of IED use will be broadly classified across four levels of the socioecological model (Stokols 1992): 1) Individual (child); 2) Interpersonal (parent/carer); 3) Environment (home, childcare and community); and 4) Policy (government). We will determine the consistency of association for each IED correlate using the model suggested by Sallis et al. 2000.

References:

Campbell M, McKenzie JE, Sowden A, Katikireddi SV, Brennan SE, Ellis S, et al. Synthesis without meta-analysis (SWiM) in systematic reviews: reporting guideline. Bmj. 2020;368:l6890.

Sallis JF, Prochaska JJ, Taylor WC. A review of correlates of physical activity of children and adolescents. Med Sci Sports Exerc. 2000;32(5):963-75.

Stokols D. Establishing and maintaining healthy environments: Toward a social ecology of health promotion. American psychologist. 1992;47(1):6.

Analysis of subgroups or subsets

Depending on sufficient reported data and the number of qualifying included studies, we will subgroup the analysis according to study design, study quality, and age group (3 years or below and 3 to 6 years) and compare studies from developed and developing countries.

REVIEW AFFILIATION, FUNDING AND PEER REVIEW

Review team members

- Professor Liane B. Azevedo, Sheffield Hallam University
- Professor Elizabeth Goyder, University of Sheffield
- Dr John Stephenson, University of Huddersfield
- Dr Daniel Jones, Teesside University
- Mr Mark Clowes, Univeristy of Sheffield
- Dr Colette Marr, Sheffield Hallam University

Review affiliation

Sheffield Hallam University

Funding source

This systematic review is funded by the National Institute for Health Research (NIHR)

Named contact

Liane B. Azevedo. Sheffield Hallam University, College of Health, Wellbeing and Life Sciences, Sheffield, S1 1WB
l.azevedo@shu.ac.uk

TIMELINE OF THE REVIEW

Review timeline

Start date: 20 May 2024. End date: 20 May 2025

Date of first submission to PROSPERO

12 May 2024

Date of registration in PROSPERO

14 May 2024

CURRENT REVIEW STAGE

Publication of review results

The intention is to publish the review once completed.The review will be published in English

Stage of the review at this submission

Review stage	Started	Completed
Pilot work		
Formal searching/study identification		
Screening search results against inclusion criteria		
Data extraction or receipt of IP		
Risk of bias/quality assessment		
Data synthesis		

Review status

The review is currently planned or ongoing.

ADDITIONAL INFORMATION

PROSPERO version history

- Version 1.0 published on 14 May 2024

Review conflict of interest

None known

Country

England

Medical Subject Headings

Child; Child Behavior; Child, Preschool; Electronics; Humans; Parents

Disclaimer

The content of this record displays the information provided by the review team. PROSPERO does not peer review registration records or endorse their content.

PROSPERO accepts and posts the information provided in good faith; responsibility for record content rests with the review team. The owner of this record has affirmed that the information provided is truthful and that they understand that deliberate provision of inaccurate information may be construed as scientific misconduct.

PROSPERO does not accept any liability for the content provided in this record or for its use. Readers use the information provided in this record at their own risk.

Any enquiries about the record should be referred to the named review contact
