# Supplementary material for: Correlates of mobile device use in young children: a systematic review and meta-analysis
Source: BMJ Public Health. 2026 Jun 17;4(2):e004305. doi: 10.1136/bmjph-2025-004305 (PMC13289221; doi:10.1136/bmjph-2025-004305)
Supplement: online supplemental file 3 [file bmjph-4-2-s003.docx]

# **Supplementary File 3**

**Search Terms Concepts and Search Strategies**

## **Search Strategy**

Table 1 Summary of database search results and updated search following deduplication against initial searches

|  | **Initial search** | | **Updated search** | |
| --- | --- | --- | --- | --- |
| **Database and platform** | **Date searched** | **Number of results** | **Date searched** | **Number of results** |
| MEDLINE (via Ovid) | 19/06/24 | 2,823 | 02/03/26 | 733 |
| Embase (via Ovid) | 19/06/24 | 2,484 | 02/03/26 | 865 |
| PsycINFO (via Ovid) | 19/06/24 | 1,365 | 02/03/26 | 237 |
| CINAHL (via EBSCOhost) | 17/06/24 | 1,134 | 02/03/26 | 249 |
| Web of Science | 19/06/24 | 2,842 | 06/03/26 | 1148 |
| Scopus | 17/06/24 | 1,651 | 05/03/26 | 478 |
| Applied Social Science Index and Abstracts - ASSIA; International Bibliography of the Social Sciences – IBSS (searched simultaneously via ProQuest) | 19/06/24 | 1,219 | 06/03/26 | 280 |
| ERIC (via EBSCOhost) | 20/06/24 | 656 | 02/03/26 | 105 |
| **Total number of results** |  | **14,180** | - | **4095** |
| **Total number of unique references after duplicates removed** |  | **7,330** |  | **9,453** |

## *Search strategy per database*

**Ovid MEDLINE (ALL)  – run 19th June 2024**

1         Child/ or child*.mp. or Child, Preschool/ 2848822

2         Infant/ or (infant or infants or kid* or toddler*).mp.      2383824

3         (nurser* or kindergarten* or daycare or ECEC or "early years" or preschool or pre-school).mp.  1033791

4         1 or 2 or 3       4373460

5         (Smartphone* or smart phone*).mp. or Cell Phones/ or (cellphone* or cell* phone*).mp.          41402

6         iPhone*.mp.  1333

7         Computers, Handheld/ or ((handheld or hand-held) adj2 (device* or computer* or phone* or technolog*)).mp.            7294

8         (mobile adj2 (device* or phone* or screen*)).mp.         21917

9         ((tablet* adj2 (digital or electronic)) or iPad*).mp.         2500

10       (touchscreen* or touch screen*).mp.  2941

11       interactive electronic device*.mp.         6

12       5 or 6 or 7 or 8 or 9 or 10 or 11  64377

13       4 and 12          7248

14       (determinant* or correlat* or predict* or "associat* with" or "associat* between" or "relat* to" or "relationship* with" or "relationship* between").mp.  12765145

15       Adolescent/ or Adult/ or (adolescen* or teen*).ti,hw,kw. 6379678

16       exp Child/ or exp Infant/ or (child* or infant*).ti,hw,kw.   3101051

17       15 not 16         4992742

18       (mhealth or m-health or telehealth or tele-health or telemedicine or tele-medicine or ((digital or mobile or online or smartphone) adj3 (survey or questionnaire* or intervention* or solution*))).mp.         142168

19       4 and 12 and 14 3911

20       19 not 17         3616

21       20 not 18         2890

22       limit 21 to english language     2823

**Embase <1974 to 2024 Week 24> - run 19^th^ June 2024**

1         child/ or preschool child/ or toddler/   2363063

2         infant/  722241

3         (child* or infant or infants or kid* or toddler*).mp.       5177801

4         (nurser* or kindergarten* or daycare or ECEC or "early years" or preschool or pre-school).mp.  680019

5         1 or 2 or 3 or 4  5190646

6         exp mobile phone/      52270

7         (smartphone* or smart phone* or cell phone* or cell* phone* or iphone*).mp.  50532

8         laptop/ 1983

9         tablet computer/          3853

10       ((handheld or hand-held) adj2 (device* or computer* or phone* or technolog*)).mp.   6011

11       (mobile adj2 (device* or phone* or screen*)).mp.         38070

12       ((tablet* adj2 (digital or electronic)) or iPad*).mp.         5097

13       (touchscreen* or touch screen*).mp.  4035

14       interactive electronic device*.mp.         10

15       6 or 7 or 8 or 9 or 10 or 11 or 12 or 13 or 14     91717

16       5 and 15          12007

17       (determinant* or correlat* or predict* or "associat* with" or "associat* between" or "relat* to" or "relationship* with" or "relationship* between").mp.  15818405

18       adolescent/    1846660

19       adult/ 9728458

20       (adolescen* or teen*).ti,hw,kw.  1912376

21       18 or 19 or 20 10509919

22       exp child/         3208760

23       exp infant/      1173704

24       (child* or infant*).ti,hw,kw.     3276116

25       22 or 23 or 24 3642829

26       21 not 25         9032262

27       (mhealth or m-health or telehealth or tele-health or telemedicine or tele-medicine or ((digital or mobile or online or smartphone) adj3 (survey or questionnaire* or intervention* or solution*))).mp.         185341

28       5 and 15 and 17 6457

29       28 not 26         5756

30       29 not 27         4822

31       limit 30 to english language     4720

32       limit 31 to conference abstracts 1326

33       31 not 32         3394

34       limit 33 to embase       2484

**APA PsycInfo <1806 to June Week 2 2024> - run 19^th^ June 2024**

1         (child* or infant or infants or kid* or toddler*).mp.       986427

2         (nurser* or kindergarten* or daycare or ECEC or "early years" or preschool or pre- school).mp. 141797

3         child day care/   2605

4         exp preschool students/ 14320

5         kindergarten students/  6680

6         1 or 2 or 3 or 4 or 5     996694

7         (smartphone* or smart phone* or cellphone* or cellphone*).mp.          9597

8         iphone*.mp.  432

9         exp mobile devices/    12418

10       ((handheld or hand-held) adj2 (device* or computer* or phone* or technolog*)).mp.   1602

11       (mobile adj2 (device* or phone* or screen*)).mp.         13098

12       ((tablet* adj2 (digital or electronic)) or iPad*).mp.         1649

13       (touchscreen* or touch screen*).mp.  1892

14       interactive electronic device*.mp.         2

15       7 or 8 or 9 or 10 or 11 or 12 or 13 or 14  24655

16       6 and 15          3515

17       (determinant* or correlat* or predict* or "associat* with" or "associat* between" or "relat* to" or "relationship* with" or "relationship* between").mp.  2858133

18       (adult* or adolescen* or teen*).ti,hw. 377764

19       (child* or infant*).ti,hw. 542681

20       18 not 19         301860

21       (mhealth or m-health or telehealth or tele-health or telemedicine or tele-medicine or ((digital or mobile or online or smartphone) adj3 (survey or questionnaire* or intervention* or solution*))).mp.         59623

22       6 and 15 and 17 1971

23       22 not 20         1701

24       23 not 21         1442

25       limit 24 to english language     1365

**CINAHL – run 17^th^ June 2024**

| **#** | **Query** | **Limiters/Expanders** | **Last Run Via** | **Results** |
| --- | --- | --- | --- | --- |
| S1 | (MH "Child") OR (MH "Child, Preschool") OR (MH "Infant") | Expanders - Apply equivalent subjects  Search modes - Boolean/Phrase | Interface - EBSCOhost Research Databases  Search Screen - Advanced Search  Database - CINAHL | 663,060 |
| S2 | child* OR infant OR infants OR kid* OR toddler* | Expanders - Apply equivalent subjects  Search modes - Boolean/Phrase | Interface - EBSCOhost Research Databases  Search Screen - Advanced Search  Database - CINAHL | 1,165,215 |
| S3 | (MH "Child Day Care") | Expanders - Apply equivalent subjects  Search modes - Boolean/Phrase | Interface - EBSCOhost Research Databases  Search Screen - Advanced Search  Database - CINAHL | 2,826 |
| S4 | nurser* OR kindergarten* OR daycare OR ECEC OR "early years" OR preschool OR pre-school | Expanders - Apply equivalent subjects  Search modes - Boolean/Phrase | Interface - EBSCOhost Research Databases  Search Screen - Advanced Search  Database - CINAHL | 243,853 |
| S5 | S1 OR S2 OR S3 OR S4 | Expanders - Apply equivalent subjects  Search modes - Boolean/Phrase | Interface - EBSCOhost Research Databases  Search Screen - Advanced Search  Database - CINAHL | 1,168,832 |
| S6 | (MH "Cellular Phone") OR (MH "Smartphone") | Expanders - Apply equivalent subjects  Search modes - Boolean/Phrase | Interface - EBSCOhost Research Databases  Search Screen - Advanced Search  Database - CINAHL | 7,157 |
| S7 | smartphone* OR "smart phone*" OR cellphone* OR "cell* phone*" OR iphone* | Expanders - Apply equivalent subjects  Search modes - Boolean/Phrase | Interface - EBSCOhost Research Databases  Search Screen - Advanced Search  Database - CINAHL | 15,498 |
| S8 | (MH "Computers, Portable+") | Expanders - Apply equivalent subjects  Search modes - Boolean/Phrase | Interface - EBSCOhost Research Databases  Search Screen - Advanced Search  Database - CINAHL | 10,569 |
| S9 | ((handheld OR hand-held) N2 (device* OR computer* OR phone* OR technolog*)) | Expanders - Apply equivalent subjects  Search modes - Boolean/Phrase | Interface - EBSCOhost Research Databases  Search Screen - Advanced Search  Database - CINAHL | 5,444 |
| S10 | (mobile N2 (device* OR phone* OR screen*)) | Expanders - Apply equivalent subjects  Search modes - Boolean/Phrase | Interface - EBSCOhost Research Databases  Search Screen - Advanced Search  Database - CINAHL | 6,785 |
| S11 | (tablet* N2 (digital OR electronic)) | Expanders - Apply equivalent subjects  Search modes - Boolean/Phrase | Interface - EBSCOhost Research Databases  Search Screen - Advanced Search  Database - CINAHL | 225 |
| S12 | touchscreen* OR "touch screen*" | Expanders - Apply equivalent subjects  Search modes - Boolean/Phrase | Interface - EBSCOhost Research Databases  Search Screen - Advanced Search  Database - CINAHL | 897 |
| S13 | interactive electronic device* | Expanders - Apply equivalent subjects  Search modes - Boolean/Phrase | Interface - EBSCOhost Research Databases  Search Screen - Advanced Search  Database - CINAHL | 4 |
| S14 | S6 OR S7 OR S8 OR S9 OR S10 OR S11 OR S12 OR S13 | Expanders - Apply equivalent subjects  Search modes - Boolean/Phrase | Interface - EBSCOhost Research Databases  Search Screen - Advanced Search  Database - CINAHL | 27,121 |
| S15 | determinant* OR correlat* OR predict* OR "associat* with" OR "associat* between" OR "relat* to" OR "relationship* with" OR "relationship between" | Expanders - Apply equivalent subjects  Search modes - Boolean/Phrase | Interface - EBSCOhost Research Databases  Search Screen - Advanced Search  Database - CINAHL | 2,359,306 |
| S16 | (MH "Adolescence") OR (MH "Adult") | Expanders - Apply equivalent subjects  Search modes - Boolean/Phrase | Interface - EBSCOhost Research Databases  Search Screen - Advanced Search  Database - CINAHL | 1,591,905 |
| S17 | TI ( adolescen* OR teen* ) OR MW ( adolescen* OR teen* ) | Expanders - Apply equivalent subjects  Search modes - Boolean/Phrase | Interface - EBSCOhost Research Databases  Search Screen - Advanced Search  Database - CINAHL | 641,718 |
| S18 | S16 OR S17 | Expanders - Apply equivalent subjects  Search modes - Boolean/Phrase | Interface - EBSCOhost Research Databases  Search Screen - Advanced Search  Database - CINAHL | 1,619,992 |
| S19 | (MH "Child+") OR (MH "Infant+") | Expanders - Apply equivalent subjects  Search modes - Boolean/Phrase | Interface - EBSCOhost Research Databases  Search Screen - Advanced Search  Database - CINAHL | 763,001 |
| S20 | TI ( child* OR infant* ) OR MW ( child* OR infant* ) | Expanders - Apply equivalent subjects  Search modes - Boolean/Phrase | Interface - EBSCOhost Research Databases  Search Screen - Advanced Search  Database - CINAHL | 941,068 |
| S21 | S19 OR S20 | Expanders - Apply equivalent subjects  Search modes - Boolean/Phrase | Interface - EBSCOhost Research Databases  Search Screen - Advanced Search  Database - CINAHL | 941,068 |
| S22 | S18 NOT S21 | Expanders - Apply equivalent subjects  Search modes - Boolean/Phrase | Interface - EBSCOhost Research Databases  Search Screen - Advanced Search  Database - CINAHL | 1,259,774 |
| S23 | S5 AND S14 AND S15 | Expanders - Apply equivalent subjects  Search modes - Boolean/Phrase | Interface - EBSCOhost Research Databases  Search Screen - Advanced Search  Database - CINAHL | 1,525 |
| S24 | ( mhealth OR m-health OR telehealth OR tele-health OR telemedicine OR tele-medicine ) OR ( ((digital OR mobile OR online OR smartphone) N3 (survey OR questionnaire* OR intervention* OR solution*)) ) | Expanders - Apply equivalent subjects  Search modes - Boolean/Phrase | Interface - EBSCOhost Research Databases  Search Screen - Advanced Search  Database - CINAHL | 76,794 |
| S25 | S23 NOT S22 | Expanders - Apply equivalent subjects  Search modes - Boolean/Phrase | Interface - EBSCOhost Research Databases  Search Screen - Advanced Search  Database - CINAHL | 1,386 |
| S26 | S25 NOT S24 | Limiters - English Language  Expanders - Apply equivalent subjects  Search modes - Boolean/Phrase | Interface - EBSCOhost Research Databases  Search Screen - Advanced Search  Database – CINAHL | 1,134 |

**Web of Science – run 19^th^ June 2024**

# Database: Web of Science Core Collection

# Searches:

1: child* or infant or infants or kid* or toddler*  (Topic) OR nurser* or kindergarten* or daycare or ECEC or "early years" or pre school or preschool  (Topic) Editions: WOS.SCI,WOS.SSCI,WOS.AHCI,WOS.ESCI,WOS.CCR,WOS.IC Date Run: Wed Jun 19 2024 09:22:07 GMT+0100 (British Summer Time) Results: 3471909

2: smartphone* or "smart phone*" or cellphone* or "cell* phone*" or iphone*  (Topic) Editions: WOS.SCI,WOS.SSCI,WOS.AHCI,WOS.ESCI,WOS.CCR,WOS.IC Date Run: Wed Jun 19 2024 09:22:37 GMT+0100 (British Summer Time) Results: 69584

3: (handheld or hand-held) NEAR/2 (device* or computer* or phone* or technolog*)  (Topic) OR mobile NEAR/2 (device* or phone* or screen*)  (Topic) OR tablet* NEAR/2 (digital or electronic)  (Topic) OR ipad*  (Topic) OR touchscreen* or "touch screen*"  (Topic) OR "interactive electronic device*"  (Topic) Editions: WOS.SCI,WOS.SSCI,WOS.AHCI,WOS.ESCI,WOS.CCR,WOS.IC Date Run: Wed Jun 19 2024 09:24:26 GMT+0100 (British Summer Time) Results: 76678

4: #2 OR #3 Editions: WOS.SCI,WOS.SSCI,WOS.AHCI,WOS.ESCI,WOS.CCR,WOS.IC Date Run: Wed Jun 19 2024 09:26:37 GMT+0100 (British Summer Time) Results: 132589

5: determinant* or correlat* or predict*  (Topic) OR "associat* with" or "associat* between"  (Topic) OR "relat* to" or "relationship* with" or "relationship* between"  (Topic) Editions: WOS.SCI,WOS.SSCI,WOS.AHCI,WOS.ESCI,WOS.CCR,WOS.IC Date Run: Wed Jun 19 2024 09:27:28 GMT+0100 (British Summer Time) Results: 15494020

6: adult* or adolescen* or teen*  (Title) OR adult* or adolescen* or teen*  (Author Keywords) Editions: WOS.SCI,WOS.SSCI,WOS.AHCI,WOS.ESCI,WOS.CCR,WOS.IC Date Run: Wed Jun 19 2024 09:28:25 GMT+0100 (British Summer Time) Results: 1022980

7: child* or infant*  (Title) OR child* or infant*  (Author Keywords) Editions: WOS.SCI,WOS.SSCI,WOS.AHCI,WOS.ESCI,WOS.CCR,WOS.IC Date Run: Wed Jun 19 2024 09:28:37 GMT+0100 (British Summer Time) Results: 1497461

8: #6 NOT #7 Editions: WOS.SCI,WOS.SSCI,WOS.AHCI,WOS.ESCI,WOS.CCR,WOS.IC Date Run: Wed Jun 19 2024 09:28:57 GMT+0100 (British Summer Time) Results: 882701

9: mhealth or m-health or telehealth or tele-health or telemedicine or tele-medicine  (Topic) OR (digital or mobile or online or smartphone) NEAR/3 (survey or questionnaire* or intervention* or solution*)  (Topic) Editions: WOS.SCI,WOS.SSCI,WOS.AHCI,WOS.ESCI,WOS.CCR,WOS.IC Date Run: Wed Jun 19 2024 09:29:36 GMT+0100 (British Summer Time) Results: 203584

10: #1 AND #4 AND #5 Editions: WOS.SCI,WOS.SSCI,WOS.AHCI,WOS.ESCI,WOS.CCR,WOS.IC Date Run: Wed Jun 19 2024 09:29:59 GMT+0100 (British Summer Time) Results: 4274

11: #10 NOT #8 Editions: WOS.SCI,WOS.SSCI,WOS.AHCI,WOS.ESCI,WOS.CCR,WOS.IC Date Run: Wed Jun 19 2024 09:30:14 GMT+0100 (British Summer Time) Results: 3618

12: #11 NOT #9 Editions: WOS.SCI,WOS.SSCI,WOS.AHCI,WOS.ESCI,WOS.CCR,WOS.IC Date Run: Wed Jun 19 2024 09:30:28 GMT+0100 (British Summer Time) Results: 2980

13: #11 NOT #9 and English (Languages) Editions: WOS.SCI,WOS.SSCI,WOS.AHCI,WOS.ESCI,WOS.CCR,WOS.IC Date Run: Wed Jun 19 2024 09:30:47 GMT+0100 (British Summer Time) Results: 2880

14: #11 NOT #9 and English (Languages) and Proceeding Paper or Meeting Abstract (Exclude – Document Types) Editions: WOS.SCI,WOS.SSCI,WOS.AHCI,WOS.ESCI,WOS.CCR,WOS.IC Date Run: Wed Jun 19 2024 09:31:37 GMT+0100 (British Summer Time) Results: 2842

**Scopus – run 17^th^ June 2024**

Advanced query

( ( ( TITLE-ABS-KEY ( child* OR infant OR infants OR kid* OR toddler* OR nurser* OR kindergarten* OR daycare OR ecec OR "early years" OR preschool OR "pre-school" ) AND TITLE-ABS-KEY ( smartphone* OR "smart phone*" OR cellphone* OR "cell* phone*" OR iphone* OR ( ( handheld OR "hand-held" ) W/3 ( phone* OR device* OR computer* OR technolog* ) ) OR "mobile device*" OR "mobile phone*" OR "mobile screen*" OR "electronic tablet*" OR "digital tablet*" OR "touch screen" OR touchscreen OR ipad* OR "interactive electronic device*" ) AND TITLE-ABS-KEY ( determinant* OR correlat* OR predict* OR "associat* with" OR "associat* between" OR "relat* to" OR "relationship* with" OR "relationship* between" ) ) ) AND NOT ( ( ( TITLE ( adult* OR adolescen* OR teen* ) OR KEY ( adult* OR adolescen* OR teen* ) ) ) AND NOT ( ( TITLE ( child* OR infant* ) OR KEY ( child* OR infant* ) ) ) ) ) AND NOT ( ( TITLE-ABS-KEY ( mhealth OR m-health OR telehealth OR "tele-health" OR telemedicine OR "tele-medicine" ) OR TITLE-ABS-KEY ( ( digital OR mobile OR online OR smartphone ) W/3 ( survey OR questionnaire* OR intervention* OR solution* ) ) ) ) AND ( LIMIT-TO ( SRCTYPE , "j" ) ) AND ( EXCLUDE ( DOCTYPE , "cp" ) OR EXCLUDE ( DOCTYPE , "ch" ) OR EXCLUDE ( DOCTYPE , "cr" ) OR EXCLUDE ( DOCTYPE , "bk" ) OR EXCLUDE ( DOCTYPE , "ed" ) OR EXCLUDE ( DOCTYPE , "re" ) OR LIMIT-TO ( DOCTYPE , "ar" ) ) AND ( LIMIT-TO ( LANGUAGE , "English" ) ) AND ( EXCLUDE ( EXACTKEYWORD , "Adolescent" ) OR EXCLUDE ( EXACTKEYWORD , "Adult" ) OR EXCLUDE ( EXACTKEYWORD , "Young Adult" ) OR EXCLUDE ( EXACTKEYWORD , "Middle Aged" ) OR EXCLUDE ( EXACTKEYWORD , "Aged" ) )

= 1,651 results

**ProQuest Social Science Collection (including IBSS and ASSIA) – run 19^th^ June 2024**

((noft(child* OR infant OR infants OR kid* OR toddler* OR nurser* OR kindergarten* OR daycare OR ECEC OR "early years" OR preschool OR pre-school) AND noft(smartphone* OR "smart phone*" OR cellphone* OR "cell* phone*" OR iphone* OR ((handheld OR hand-held) NEAR/2 (device* OR computer* OR phone* OR technolog*)) OR (mobile NEAR/2 (device* OR phone* OR screen*)) OR (tablet* NEAR/2 (digital OR electronic)) OR (touchscreen* OR "touch screen*") OR "interactive electronic device*") AND noft(determinant* OR correlat* OR predict* OR "associat* with" OR "associat* between" OR "relat* to" OR "relationship* with" OR "relationship between")) NOT ((title(adolescen* OR teen*) OR subject(adolescen* OR teen*)) NOT (title(child* OR infant*) OR subject(child* OR infant*)))) NOT (noft(mhealth OR m-health OR telehealth OR tele-health OR telemedicine OR tele-medicine) OR noft((digital OR mobile OR online OR smartphone) NEAR/3 (survey OR questionnaire* OR intervention* OR solution*)))

= 1,219 results

**ERIC – run 20th June 2024**

| **#** | **Query** | **Limiters/Expanders** | **Last Run Via** | **Results** |
| --- | --- | --- | --- | --- |
| S5 | S1 NOT (S2 OR S3) | Expanders - Apply equivalent subjects  Narrow by Language: - english  Search modes - Boolean/Phrase | Interface - EBSCOhost Research Databases  Search Screen - Advanced Search  Database - ERIC | **656** |
| S4 | S1 NOT (S2 OR S3) | Expanders - Apply equivalent subjects  Search modes - Boolean/Phrase | Interface - EBSCOhost Research Databases  Search Screen - Advanced Search  Database - ERIC | 863 |
| S3 | ( mhealth OR m-health OR telehealth OR tele-health OR telemedicine OR tele-medicine ) OR ( ((digital OR mobile OR online OR smartphone) N3 (survey OR questionnaire* OR intervention* OR solution*)) ) | Expanders - Apply equivalent subjects  Search modes - Boolean/Phrase | Interface - EBSCOhost Research Databases  Search Screen - Advanced Search  Database - ERIC | 14,861 |
| S2 | ( TI ( Adolescen* OR adult* OR teen* ) OR TI ( Adolescen* OR adult* OR teen* ) ) NOT ( TI ( child* OR infant* ) OR SU ( child* OR infant* ) ) | Expanders - Apply equivalent subjects  Search modes - Boolean/Phrase | Interface - EBSCOhost Research Databases  Search Screen - Advanced Search  Database - ERIC | 52,965 |
| S1 | ( child* OR infant OR infants OR kid* OR toddler* OR nurser* OR kindergarten* OR daycare OR ECEC OR "early years" OR preschool OR pre-school ) AND ( smartphone* OR "smart phone*" OR cellphone* OR "cell* phone*" OR iphone* OR handheld OR hand-held OR "mobile device*" OR "mobile phone*" OR "electronic tablet*" OR "digital tablet*" OR touchscreen* OR "touch screen*" OR "interactive electronic device*" ) AND ( determinant* OR correlat* OR predict* OR "associat* with" OR "associat* between" OR "relat* to" OR "relationship* with" OR "relationship between" ) AND ( determinant* OR correlat* OR predict* OR "associat* with" OR "associat* between" OR "relat* to" OR "relationship* with" OR "relationship between" ) | Expanders - Apply equivalent subjects  Search modes - Boolean/Phrase | Interface - EBSCOhost Research Databases  Search Screen - Advanced Search  Database - ERIC | 942 |

## Search strategies – Updated search 2026

**Ovid MEDLINE(R) ALL** <1946 to February 27, 2026>

1 Child/ or child*.mp. or Child, Preschool/ 3032033

2 Infant/ or (infant or infants or kid* or toddler*).mp. 2522488

3 (nurser* or kindergarten* or daycare or ECEC or "early years" or preschool or pre-school).mp. 1093465

4 1 or 2 or 3 4648123

5 (Smartphone* or smart phone*).mp. or Cell Phones/ or (cellphone* or cell* phone*).mp. 50181

6 iPhone*.mp. 1560

7 Computers, Handheld/ or ((handheld or hand-held) adj2 (device* or computer* or phone* or technolog*)).mp. 7863

8 (mobile adj2 (device* or phone* or screen*)).mp. 25456

9 ((tablet* adj2 (digital or electronic)) or iPad*).mp. 2810

10 (touchscreen* or touch screen*).mp. 3280

11 interactive electronic device*.mp. 8

12 5 or 6 or 7 or 8 or 9 or 10 or 11 76682

13 4 and 12 8608

14 (determinant* or correlat* or predict* or "associat* with" or "associat* between" or "relat* to" or "relationship* with" or "relationship* between").mp. 14083900

15 Adolescent/ or Adult/ or (adolescen* or teen*).ti,hw,kw. 6872087

16 exp Child/ or exp Infant/ or (child* or infant*).ti,hw,kw. 3265410

17 15 not 16 5400357

18 (mhealth or m-health or telehealth or tele-health or telemedicine or tele-medicine or ((digital or mobile or online or smartphone) adj3 (survey or questionnaire* or intervention* or solution*))).mp. 178107

19 4 and 12 and 14 4768

20 19 not 17 4376

21 20 not 18 3489

22 limit 21 to english language 3415

23 limit 22 to yr="2024 -Current" **733**

**Embase** <1974 to 2026 Week 09>

1 child/ or preschool child/ or toddler/ 2654788

2 infant/ 796865

3 (child* or infant or infants or kid* or toddler*).mp. 5726580

4 (nurser* or kindergarten* or daycare or ECEC or "early years" or preschool or pre-school).mp. 733117

5 1 or 2 or 3 or 4 5741024

6 exp mobile phone/ 70649

7 (smartphone* or smart phone* or cell phone* or cell* phone* or iphone*).mp. 68764

8 laptop/ 3519

9 tablet computer/ 5720

10 ((handheld or hand-held) adj2 (device* or computer* or phone* or technolog*)).mp. 7584

11 (mobile adj2 (device* or phone* or screen*)).mp. 46666

12 ((tablet* adj2 (digital or electronic)) or iPad*).mp. 6492

13 (touchscreen* or touch screen*).mp. 4760

14 interactive electronic device*.mp. 13

15 6 or 7 or 8 or 9 or 10 or 11 or 12 or 13 or 14 120111

16 5 and 15 16597

17 (determinant* or correlat* or predict* or "associat* with" or "associat* between" or "relat* to" or "relationship* with" or "relationship* between").mp. 17820980

18 adolescent/ 2056832

19 adult/ 11432196

20 (adolescen* or teen*).ti,hw,kw. 2127038

21 18 or 19 or 20 12299375

22 exp child/ 3558930

23 exp infant/ 1278215

24 (child* or infant*).ti,hw,kw. 3628459

25 22 or 23 or 24 4020975

26 21 not 25 10607166

27 (mhealth or m-health or telehealth or tele-health or telemedicine or tele-medicine or ((digital or mobile or online or smartphone) adj3 (survey or questionnaire* or intervention* or solution*))).mp. 243638

28 5 and 15 and 17 9246

29 28 not 26 8179

30 29 not 27 6720

31 limit 30 to english language 6607

32 limit 31 to conference abstracts 1528

33 31 not 32 5079

34 limit 33 to embase 3206

35 limit 34 to yr="2024 -Current" **865**

**APA PsycInfo** <1806 to February 2026 Week 4>

1 (child* or infant or infants or kid* or toddler*).mp. 1040316

2 (nurser* or kindergarten* or daycare or ECEC or "early years" or preschool or pre- school).mp. 148524

3 child day care/ 2640

4 exp preschool students/ 15852

5 kindergarten students/ 7086

6 1 or 2 or 3 or 4 or 5 1051242

7 (smartphone* or smart phone* or cellphone* or cellphone*).mp. 11875

8 iphone*.mp. 468

9 exp mobile devices/ 13777

10 ((handheld or hand-held) adj2 (device* or computer* or phone* or technolog*)).mp. 1656

11 (mobile adj2 (device* or phone* or screen*)).mp. 14496

12 ((tablet* adj2 (digital or electronic)) or iPad*).mp. 1766

13 (touchscreen* or touch screen*).mp. 2041

14 interactive electronic device*.mp. 2

15 7 or 8 or 9 or 10 or 11 or 12 or 13 or 14 28235

16 6 and 15 4085

17 (determinant* or correlat* or predict* or "associat* with" or "associat* between" or "relat* to" or "relationship* with" or "relationship* between").mp. 3064311

18 (adult* or adolescen* or teen*).ti,hw. 414487

19 (child* or infant*).ti,hw. 575862

20 18 not 19 332495

21 (mhealth or m-health or telehealth or tele-health or telemedicine or tele-medicine or ((digital or mobile or online or smartphone) adj3 (survey or questionnaire* or intervention* or solution*))).mp. 74218

22 6 and 15 and 17 2387

23 22 not 20 2046

24 23 not 21 1739

25 limit 24 to english language 1651

26 limit 25 to yr="2024 -Current" **237**

**_________________________________________________________________________________**

**CINAHL**

S28 [S25 NOT S24](https://research-ebsco-com.sheffield.idm.oclc.org/search/results?combinedSearchQueryId=sq%3A862f31b9-66f5-4ad5-9f2b-33c504933acb&db=cin20&expanders=concept&limiters=DT1%3A2024-01-01%2F2026-12-31&searchMode=boolean&sort=relevance&sqId=sq%3A862f31b9-66f5-4ad5-9f2b-33c504933acb&userDirectAction=true) Results: **249** 01/01/2024 - 12/31/2026

S27 [S25 NOT S24](https://research-ebsco-com.sheffield.idm.oclc.org/search/results?combinedSearchQueryId=sq%3A18070385-b90e-4116-aee8-4130d63bfb1b&db=cin20&expanders=concept&limiters=None&searchMode=boolean&sort=relevance&sqId=sq%3A18070385-b90e-4116-aee8-4130d63bfb1b&userDirectAction=true) Results: 1,327

S26 [S25 NOT S24](https://research-ebsco-com.sheffield.idm.oclc.org/search/results?combinedSearchQueryId=sq%3A0f6d7c8e-af80-4f67-9c95-368b39980346&db=cin20&expanders=concept&limiters=None&searchMode=boolean&sort=relevance&sqId=sq%3A0f6d7c8e-af80-4f67-9c95-368b39980346&userDirectAction=true) Results: 1,327

S25 [S23 NOT S22](https://research-ebsco-com.sheffield.idm.oclc.org/search/results?combinedSearchQueryId=sq%3Ab5e75e79-88ed-42a3-937b-9e88d687ed6a&db=cin20&expanders=concept&limiters=None&searchMode=boolean&sort=relevance&sqId=sq%3Ab5e75e79-88ed-42a3-937b-9e88d687ed6a&userDirectAction=true) Results: 1,584

S24 [( mhealth OR m-health OR telehealth OR tele-health OR telemedicine OR tele-medicine ) OR ( ((digital OR mobile OR online OR smartphone) N3 (survey OR questionnaire* OR intervention* OR solution*)) )](https://research-ebsco-com.sheffield.idm.oclc.org/search/results?db=cin20&expanders=concept&limiters=None&searchMode=boolean&sort=relevance&sqId=sq%3A975ed1c9-aaf0-48bc-96c0-f8a6a6c9d227&userDirectAction=true) Results: 91,495

S23 [S5 AND S14 AND S15](https://research-ebsco-com.sheffield.idm.oclc.org/search/results?combinedSearchQueryId=sq%3A685ede17-f182-4823-af84-ee45f3af2247&db=cin20&expanders=concept&limiters=None&searchMode=boolean&sort=relevance&sqId=sq%3A685ede17-f182-4823-af84-ee45f3af2247&userDirectAction=true) Results: 1,747

S22 [S18 NOT S21](https://research-ebsco-com.sheffield.idm.oclc.org/search/results?combinedSearchQueryId=sq%3A82b141a6-15a5-4f19-a5b4-cd5b5b684ff0&db=cin20&expanders=concept&limiters=None&searchMode=boolean&sort=relevance&sqId=sq%3A82b141a6-15a5-4f19-a5b4-cd5b5b684ff0&userDirectAction=true) Results: 1,357,478

S21 [S20 OR S19](https://research-ebsco-com.sheffield.idm.oclc.org/search/results?combinedSearchQueryId=sq%3Ace398077-d03d-47c9-a4bd-c352a2409a9a&db=cin20&expanders=concept&limiters=None&searchMode=boolean&sort=relevance&sqId=sq%3Ace398077-d03d-47c9-a4bd-c352a2409a9a&userDirectAction=true) Results: 1,024,413

S20 [TI ( child* OR infant* ) OR MW ( child* OR infant* )](https://research-ebsco-com.sheffield.idm.oclc.org/search/results?db=cin20&expanders=concept&limiters=None&searchMode=boolean&sort=relevance&sqId=sq%3A529bdec5-47c2-49c4-a49f-a7aca9668fa5&userDirectAction=true) Results: 1,024,413

S19 [(MH "Child+") OR (MH "Infant+")](https://research-ebsco-com.sheffield.idm.oclc.org/search/results?db=cin20&expanders=concept&limiters=None&searchMode=boolean&sort=relevance&sqId=sq%3A8e721069-14f1-4c1e-a139-0bb32114e73a&userDirectAction=true) Results: 810,339

S18 [S16 OR S17](https://research-ebsco-com.sheffield.idm.oclc.org/search/results?combinedSearchQueryId=sq%3A419d8848-566e-422b-b833-89735769d61b&db=cin20&expanders=concept&limiters=None&searchMode=boolean&sort=relevance&sqId=sq%3A419d8848-566e-422b-b833-89735769d61b&userDirectAction=true) Results: 1,743,520

S17 [TI ( adolescen* OR teen* ) OR MW ( adolescen* OR teen* )](https://research-ebsco-com.sheffield.idm.oclc.org/search/results?db=cin20&expanders=concept&limiters=None&searchMode=boolean&sort=relevance&sqId=sq%3A8ae84d4b-54e8-4734-8293-f8751351f51c&userDirectAction=true) Results: 697,031

S16 [(MH "Adolescence") OR (MH "Adult")](https://research-ebsco-com.sheffield.idm.oclc.org/search/results?db=cin20&expanders=concept&limiters=None&searchMode=boolean&sort=relevance&sqId=sq%3Abcf2df0a-8c29-46e6-8fb6-eae9671d1d98&userDirectAction=true) Results: 1,708,497

S15 [determinant* OR correlat* OR predict* OR "associat* with" OR "associat* between" OR "relat* to" OR "relationship* with" OR "relationship between"](https://research-ebsco-com.sheffield.idm.oclc.org/search/results?db=cin20&expanders=concept&limiters=None&searchMode=boolean&sort=relevance&sqId=sq%3A3aa803d5-7571-499f-97c6-c130afc6f8be&userDirectAction=true) Results: 2,573,358

S14 [S6 OR S7 OR S8 OR S9 OR S10 OR S11 OR S12 OR S13](https://research-ebsco-com.sheffield.idm.oclc.org/search/results?combinedSearchQueryId=sq%3A44ddf9ba-8185-48b8-b08f-66cce570d248&db=cin20&expanders=concept&limiters=None&searchMode=boolean&sort=relevance&sqId=sq%3A44ddf9ba-8185-48b8-b08f-66cce570d248&userDirectAction=true) Results: 30,193

S13 [interactive electronic device*](https://research-ebsco-com.sheffield.idm.oclc.org/search/results?db=cin20&expanders=concept&limiters=None&searchMode=boolean&sort=relevance&sqId=sq%3Ac7652049-5eca-4ba7-b345-0b5178738ddd&userDirectAction=true) Results: 5

S12 [touchscreen* OR "touch screen*"](https://research-ebsco-com.sheffield.idm.oclc.org/search/results?db=cin20&expanders=concept&limiters=None&searchMode=boolean&sort=relevance&sqId=sq%3Afca2f2ef-927c-458d-85c9-4928e8dd698d&userDirectAction=true) Results: 954

S11 [(tablet* N2 (digital OR electronic))](https://research-ebsco-com.sheffield.idm.oclc.org/search/results?db=cin20&expanders=concept&limiters=None&searchMode=boolean&sort=relevance&sqId=sq%3A2c0785e6-304e-40c7-8e76-2e2efb0caeab&userDirectAction=true) Results: 246

S10 [(mobile N2 (device* OR phone* OR screen*))](https://research-ebsco-com.sheffield.idm.oclc.org/search/results?db=cin20&expanders=concept&limiters=None&searchMode=boolean&sort=relevance&sqId=sq%3A9e8437e5-38dd-4fa7-b845-17b78d7deb0b&userDirectAction=true) Results: 7,368

S9 [((handheld OR hand-held) N2 (device* OR computer* OR phone* OR technolog*))](https://research-ebsco-com.sheffield.idm.oclc.org/search/results?db=cin20&expanders=concept&limiters=None&searchMode=boolean&sort=relevance&sqId=sq%3Ad13d30b2-f7e3-42f6-973c-26e39825022c&userDirectAction=true)

Results: 5,614

S8 [(MH "Computers, Portable+")](https://research-ebsco-com.sheffield.idm.oclc.org/search/results?db=cin20&expanders=concept&limiters=None&searchMode=boolean&sort=relevance&sqId=sq%3Ac15d6cc8-1ce0-49d4-8e69-1328a4194517&userDirectAction=true) 12,150

S7 [smartphone* OR "smart phone*" OR cellphone* OR "cell* phone*" OR iphone*](https://research-ebsco-com.sheffield.idm.oclc.org/search/results?db=cin20&expanders=concept&limiters=None&searchMode=boolean&sort=relevance&sqId=sq%3A0ff43353-eeb7-43d9-8dc9-5ea93392d0da&userDirectAction=true) Results: 17,998

S6 [(MH "Cellular Phone") OR (MH "Smartphone")](https://research-ebsco-com.sheffield.idm.oclc.org/search/results?db=cin20&expanders=concept&limiters=None&searchMode=boolean&sort=relevance&sqId=sq%3A07650e23-59a8-4ba1-8eb8-813181c7063c&userDirectAction=true) Results: 8,862

S5 [S4 OR S3 OR S2 OR S1](https://research-ebsco-com.sheffield.idm.oclc.org/search/results?combinedSearchQueryId=sq%3Aaeeb0b07-4ac3-4e2c-be6f-2e23a456f0be&db=cin20&expanders=concept&limiters=None&searchMode=boolean&sort=relevance&sqId=sq%3Aaeeb0b07-4ac3-4e2c-be6f-2e23a456f0be&userDirectAction=true) Results: 1,280,710

S4 [nurser* OR kindergarten* OR daycare OR ECEC OR "early years" OR preschool OR pre-school](https://research-ebsco-com.sheffield.idm.oclc.org/search/results?db=cin20&expanders=concept&limiters=None&searchMode=boolean&sort=relevance&sqId=sq%3A96f93699-4364-4add-9bef-f400ddf4f28e&userDirectAction=true) Results: 258,668

S3 [(MH "Child Day Care")](https://research-ebsco-com.sheffield.idm.oclc.org/search/results?db=cin20&expanders=concept&limiters=None&searchMode=boolean&sort=relevance&sqId=sq%3A823912b1-5833-4132-99fc-768d8dfc1fd5&userDirectAction=true) Results: 2,907

S2 [child* OR infant OR infants OR kid* OR toddler*](https://research-ebsco-com.sheffield.idm.oclc.org/search/results?db=cin20&expanders=concept&limiters=None&searchMode=boolean&sort=relevance&sqId=sq%3A346bde3c-13a3-41d9-bfaa-a646e6fb51a1&userDirectAction=true) Results: 1,275,544

S1 [(MH "Child") OR (MH "Child, Preschool") OR (MH "Infant")](https://research-ebsco-com.sheffield.idm.oclc.org/search/results?db=cin20&expanders=concept&limiters=None&searchMode=boolean&sort=relevance&sqId=sq%3A8d70be9a-581e-4b1e-8b64-f3093c525cc5&userDirectAction=true) Results: 702,011

_________________________________________________________________________________

**Web of Science** (searched 6.3.26)

# Searches:

Search: #7 NOT #5 and 2024 or 2025 or 2026 (Publication Years) and English (Languages) Results: **1148**

Search: #7 NOT #5 and 2024 or 2025 or 2026 (Publication Years) Results: 1174

Search: #7 NOT #5 Results: 5275

Search: #6 NOT #4 Results: 6449

Search: #1 AND #2 AND #3 Results: 6540

Search 5: mhealth or m-health or telehealth or tele-health or telemedicine or tele-medicine (Topic) OR (digital or mobile or online or smartphone) NEAR/3 (survey or questionnaire* or intervention* or solution*) (Topic) Results: 418778

Search 4: adult* or adolescen* or teen* (Topic) NOT child* or infant* (Topic) Results: 8238760

Search 3: determinant* or correlat* or predict* (Topic) OR "associat* with" or "associat* between" (Topic) OR "relat* to" or "relationship* with" or "relationship* between" (Topic) Results: 35392612

Search 2: smartphone* or "smart phone*" or cellphone* or "cell* phone*" or iphone* (Topic) OR (handheld or hand-held) NEAR/2 (device* or computer* or phone* or technolog*) (Topic) OR mobile NEAR/2 (device* or phone* or screen*) (Topic) OR tablet* NEAR/2 (digital or electronic) (Topic) OR iPad* or touchscreen* or "touch screen*" or "interactive electronic device*" (Topic) Results: 2037689

Search 1: (child* or infant or infants or kid* or toddler*) (Topic) OR (nurser* or kindergarten* or daycare or ECEC or "early years" or "pre school" or preschool) (Topic) and Web of Science Core Collection (Database) Results: 5193817

**Scopus**

Run on 05/02/2026

( ( ( TITLE-ABS-KEY ( child* OR infant OR infants OR kid* OR toddler* OR nurser* OR kindergarten* OR daycare OR ecec OR "early years" OR preschool OR "pre-school" ) AND TITLE-ABS-KEY ( smartphone* OR "smart phone*" OR cellphone* OR "cell* phone*" OR iphone* OR ( ( handheld OR "hand-held" ) W/3 ( phone* OR device* OR computer* OR technolog* ) ) OR "mobile device*" OR "mobile phone*" OR "mobile screen*" OR "electronic tablet*" OR "digital tablet*" OR "touch screen" OR touchscreen OR ipad* OR "interactive electronic device*" ) AND TITLE-ABS-KEY ( determinant* OR correlat* OR predict* OR "associat* with" OR "associat* between" OR "relat* to" OR "relationship* with" OR "relationship* between" ) ) ) AND NOT ( ( ( TITLE ( adult* OR adolescen* OR teen* ) OR KEY ( adult* OR adolescen* OR teen* ) ) ) AND NOT ( ( TITLE ( child* OR infant* ) OR KEY ( child* OR infant* ) ) ) ) ) AND NOT ( ( TITLE-ABS-KEY ( mhealth OR m-health OR telehealth OR "tele-health" OR telemedicine OR "tele-medicine" ) OR TITLE-ABS-KEY ( ( digital OR mobile OR online OR smartphone ) W/3 ( survey OR questionnaire* OR intervention* OR solution* ) ) ) ) AND ( LIMIT-TO ( SRCTYPE , "j" ) ) AND ( LIMIT-TO ( DOCTYPE , "ar" ) OR EXCLUDE ( DOCTYPE , "cp" ) OR EXCLUDE ( DOCTYPE , "ch" ) OR EXCLUDE ( DOCTYPE , "cr" ) OR EXCLUDE ( DOCTYPE , "bk" ) OR EXCLUDE ( DOCTYPE , "ed" ) OR EXCLUDE ( DOCTYPE , "re" ) ) AND ( LIMIT-TO ( PUBYEAR , 2024 ) OR LIMIT-TO ( PUBYEAR , 2025 ) OR LIMIT-TO ( PUBYEAR , 2026 ) ) AND ( LIMIT-TO ( LANGUAGE , "English" ) ) AND ( EXCLUDE ( EXACTKEYWORD , "Adolescent" ) OR EXCLUDE ( EXACTKEYWORD , "Adult" ) OR EXCLUDE ( EXACTKEYWORD , "Young Adult" ) OR EXCLUDE ( EXACTKEYWORD , "Middle Aged" ) OR EXCLUDE ( EXACTKEYWORD , "Aged" ) )

Results = **478**

**ProQuest (including ASSIA and IBSS)**

[(((noft(child* OR infant OR infants OR kid* OR toddler* OR nurser* OR kindergarten* OR daycare OR ECEC OR "early years" OR preschool OR pre-school) AND noft(smartphone* OR "smart phone*" OR cellphone* OR "cell* phone*" OR iphone* OR ((handheld OR hand-held) NEAR/2 (device* OR computer* OR phone* OR technolog*)) OR (mobile NEAR/2 (device* OR phone* OR screen*)) OR (tablet* NEAR/2 (digital OR electronic)) OR (touchscreen* OR "touch screen*") OR "interactive electronic device*") AND noft(determinant* OR correlat* OR predict* OR "associat* with" OR "associat* between" OR "relat* to" OR "relationship* with" OR "relationship between")) NOT ((title(adolescen* OR teen*) OR subject(adolescen* OR teen*)) NOT (title(child* OR infant*) OR subject(child* OR infant*)))) NOT (noft(mhealth OR m-health OR telehealth OR tele-health OR telemedicine OR tele-medicine) OR noft((digital OR mobile OR online OR smartphone) NEAR/3 (survey OR questionnaire* OR intervention* OR solution*)))) NOT (bdl(1007535) AND pd(20240101-20260306))](https://www.proquest.com/myresearch/savedsearches.checkdbssearchlink:rerunsearch/3038330/SavedSearches/$N?_csrf=27247d06-569a-4599-a71d-4a93ab16087f&site=socialsciencepremium&t:ac=SavedSearches)

Results = **280**

**ERIC**

S6 [S1 NOT (S2 OR S3)](https://research-ebsco-com.sheffield.idm.oclc.org/search/results?combinedSearchQueryId=sq%3A11d36045-141b-4569-be14-fe9e2af5cc62&db=eric&expanders=concept&limiters=DT1%3A2024-01-01%2F2026-12-31&searchMode=all&sort=relevance&sqId=sq%3A11d36045-141b-4569-be14-fe9e2af5cc62&userDirectAction=true) Results: **105** (01/01/2024 - 12/31/2026)

S5 [S1 NOT (S2 OR S3)](https://research-ebsco-com.sheffield.idm.oclc.org/search/results?combinedSearchQueryId=sq%3A282b9f5a-387e-47db-9043-3076813315be&db=eric&expanders=concept&limiters=None&searchMode=all&sort=relevance&sqId=sq%3A282b9f5a-387e-47db-9043-3076813315be&userDirectAction=true) Results: 961

S4 [S1 NOT (S2 OR S3)](https://research-ebsco-com.sheffield.idm.oclc.org/search/results?combinedSearchQueryId=sq%3A307eef96-0634-4132-9993-912a31cad3f6&db=eric&expanders=concept&limiters=None&searchMode=all&sort=relevance&sqId=sq%3A307eef96-0634-4132-9993-912a31cad3f6&userDirectAction=true) Results: 961

S3 [( mhealth OR m-health OR telehealth OR tele-health OR telemedicine OR tele-medicine ) OR ( ((digital OR mobile OR online OR smartphone) N3 (survey OR questionnaire* OR intervention* OR solution*)) )](https://research-ebsco-com.sheffield.idm.oclc.org/search/results?db=eric&expanders=concept&limiters=None&searchMode=all&sort=relevance&sqId=sq%3Afd3121d5-2687-4da8-b61a-c89ef38c1910&userDirectAction=true) Results: 17,488

S2 [( TI ( Adolescen* OR adult* OR teen* ) OR TI ( Adolescen* OR adult* OR teen* ) ) NOT ( TI ( child* OR infant* ) OR SU ( child* OR infant* ) )](https://research-ebsco-com.sheffield.idm.oclc.org/search/results?db=eric&expanders=concept&limiters=None&searchMode=all&sort=relevance&sqId=sq%3A222958a1-a01c-4ff0-987a-6daac1e3a569&userDirectAction=true) Results: 55,410

S1 [( child* OR infant OR infants OR kid* OR toddler* OR nurser* OR kindergarten* OR daycare OR ECEC OR "early years" OR preschool OR pre-school ) AND ( smartphone* OR "smart phone*" OR cellphone* OR "cell* phone*" OR iphone* OR handheld OR hand-held OR "mobile device*" OR "mobile phone*" OR "electronic tablet*" OR "digital tablet*" OR touchscreen* OR "touch screen*" OR "interactive electronic device*" ) AND ( determinant* OR correlat* OR predict* OR "associat* with" OR "associat* between" OR "relat* to" OR "relationship* with" OR "relationship between" ) AND ( determinant* OR correlat* OR predict* OR "associat* with" OR "associat* between" OR "relat* to" OR "relationship* with" OR "relationship between" )](https://research-ebsco-com.sheffield.idm.oclc.org/search/results?db=eric&expanders=concept&limiters=None&searchMode=all&sort=relevance&sqId=sq%3A2d3c8155-18d4-4a21-9da1-af4a82ea0475&userDirectAction=true) Results:

1,056
