# Supplementary material for: Correlates of mobile device use in young children: a systematic review and meta-analysis
Source: BMJ Public Health. 2026 Jun 17;4(2):e004305. doi: 10.1136/bmjph-2025-004305 (PMC13289221; doi:10.1136/bmjph-2025-004305)
Supplement: online supplemental file 4 [file bmjph-4-2-s004.docx]

**Supplementary File 4**

**Derivation of effect statistics and Fisher’s Z transformation**

1. **Derivation of effect statistics**

For studies which expressed the relationship between the outcome (*y*) and the correlate (*x*) in terms of the covariance between *y* and *x*, the correlation coefficient was evaluated using the formula

$$r=\frac{Cov(x,y)}{\sigma_{x}\cdot\sigma_{y}}$$

For studies which expressed the relationship between the outcome and the correlate in terms of the χ^2^ statistic (with 1 degree of freedom), the φ statistic was derived using the following expression:

$$\phi=\sqrt{\frac{\chi^{2}}{n}}$$

where *n* is the sample size. A variant of the φ statistic, Cramer’s *V* statistic was derived for some studies with multiple categories of the correlate but was in all cases identical to the corresponding φ statistic.

For studies which expressed the relationship between PSU and the correlate in terms of odds ratio (OR), the φ statistic was derived using the following expression:

$$\phi=\frac{ln(OR)}{\sqrt{\left[ \left[ {ln(OR)}^{2} \right]+\frac{\pi^{2}}{9} \right]}}$$

For studies which expressed the relationship between PSU and the correlate in terms of the *t*-statistic, the correlation coefficient *r* was derived using the following expression:

$$r=\frac{t}{\sqrt{t^{2}+df}}$$

where *df* is the degrees of freedom, given by $df=n-2$ for a study of size *n*.

Studies that reported correlation coefficients, φ-statistics or standardised regression coefficients in simple linear regression were carried forward to the next stage of the process without further transformation.

1. **Fisher’s Z transformation**

Fisher’s *Z* transformation was used for this process, given by the following expression:

$$Z=\frac{1}{2}ln\frac{1+r}{1-r}$$

The standard error of the Fisher-transformed coefficient, SE(Z), is given by:

$$SE\left( Z \right)=\frac{1}{\sqrt{n-3}}$$

Following meta-analyses, the synthesised estimate of the correlation coefficient (and corresponding lower and upper 95% confidence intervals (CIs)) was obtained using the following expression:

$$r=\frac{e^{2Z}-1}{e^{2Z}+1}$$
