# Supplementary material for: Correlates of mobile device use in young children: a systematic review and meta-analysis
Source: BMJ Public Health. 2026 Jun 17;4(2):e004305. doi: 10.1136/bmjph-2025-004305 (PMC13289221; doi:10.1136/bmjph-2025-004305)
Supplement: online supplemental file 5 [file bmjph-4-2-s005.docx]

**Supplementary File 5.** Summary of the included studies

| **Author year; country; study design** | **Participants demographic (sample, age, sex, SES)** | **Mobile device exposure (e.g., type and duration, content, reporting type)** | **Correlated of mobile device use– reported by socio-ecological level and type of devices** | **Author’s conclusion regarding the correlates** |
| --- | --- | --- | --- | --- |
| Abdullah et al. 2022;^54^ Malaysia; Cross-sectional | N= 364  Age: range 5 to 6 years  Sex: 48.4% male  SES:  Household income: Top 20 (33.2%), Middle 40 (36%), Bottom 40 (30.8%)  Level of education: Certification (25.0%), Diploma (21.4%), Bachelor's degree (35.7%), Master's/doctoral degree (17.9%) | Type and duration: Smartphones use: 72.8%  Tablets use: 27.2%  Content: 80.5% entertainment purposes (e.g., games, videos, web surfing, listening to music); 19.5% educational purposes​.  Reporting type: parent-reported questionnaire adapted from the Problematic Mobile Phone Use Scale^56^ | *Individual (PSU):*  Sex (ref girls)  *Interpersonal (PSU):*  Parent's education (ref Masters/doctoral); Household income (ref income top 40%); Purposes of providing mobile devices to the children (ref: sit still) | Sex, the age of children when starting to use mobile devices, and the purpose of providing mobile devices to children significantly contributed to excessive mobile use among young children. |
| Abdullah et al. 2023;^59^ Bangladesh; Cross-sectional | N=400  Age range: 5 to 6 years  Sex: not reported  SES: not reported | Type and duration:  Use smartphone less than 1 hour 36 % (n=144)  Use smartphone more than 1 hour 64% (n=256)  Content:  79% cartoon/fiction; 49% game; 45.3 % TV/Video/Music; 13.8% Study  Reporting type: Problematic smartphone use score was categorized according to previous studies.^56,57,58^  Scores were made by using self-judgment. | *Individual (PSU):*  Child Age (3, 4,5)  *Interpersonal (PSU):*  Mother's daily use of smartphone < = 3 hours vs >3 hrs; Father's daily use of smartphone (>3 hours <3 hours); Mother's education level (Prim/sec/above); Father's education level (Prim/sec/above); Mother's profession (Housewife/worker/pro); Father's profession (worker/prof/business); Family income (<15K, 15-25K, >25K); Mother Age (18-25, 26-30, 31+); Number of siblings (1, 2, 3+) | The likelihood of PSU is linked with various factors such as parental attributes like daily usage of smartphone, education level, profession, family income, age of mother. |
| Ali et al., 2022;^61^  Jordan; cross-sectional | n = 2,781  Age range: less than 6 years  Sex: 53.1% male (n=1,477)  SES:  Mothers' education: Primary or lower = 0.3% (8), High school = 14.9%(413), Diploma = 13.6%(378), Bachelor = 59.9%(1667), Higher education = 11.3%(315)  Fathers' education: Primary or lower =1.6%(44), High school = 28.7%(798), Diploma = 11.9%(332), Bachelor = 43.9%(1220), Higher education = 13.9%(387)  Family income (JD/month) ≤450 JD = 34.9% (933) 451–650 JD = 25.0% (668) 651–850 JD = 15.0% (400) 851–1,050 JD =13.8% (368) >1,050 JD = 11.3 (301) | Type and duration:  Use of smartphone before pandemic: 1.67 (1.59) hours per day  Content: not reported  Reporting type: Customised self-reporting questionnaire | *Interpersonal (smartphone only):*  Parental knowledge of smartphone exposure; Parents daily smartphone use | Parents were generally knowledgeable about the effects of smartphone exposure on children's health, though there were knowledge gaps concerning parental controls and the safe levels of smartphone exposure for infants. |
| Bui et al. 2022;^58^  Australia; cross-sectional | N=214  Age: 5.23 (0.44) years  Sex: 62.6% male  SES:  Education  0.5% less than 10 year Cert, 9.8% High school certificate, 21.5% Dip/TAFE equivalent, 36% undergraduate degree, 32.2% postgraduate degree.  Income  28.5% <$100,000, 29.9% $100,000-150,000, 20.6% $150,000-$200,000, 21% >$200,000 | Type and duration:  Tablet/iPad: n= 184 (86%); Smartphone: n= 5 (2.3%)  Content:  Touch/scroll to look at things: n= 169 (79%); Watch videos: n= 181 (84.6%);  Reporting type:  Common Sense Media’s Screen Time adapted  survey (Rideout, 2013) responded by parents -how much time  in hours they spend with handheld devices (iPad, tablet, smartphones or similar) | *Individual (smartphone and tablet separate):*  Child internalising problems; Child externalising problems; Child externalising problems  *Interpersonal (smartphone and tablet separate):*  Parental anxiety; Parental depression; Parental stress; Parental phone use; Parental tablet use; Parental age; Annual household income; Highest education level | The mental health of parents has an effect on the amount of screen time in children.  Parental anxiety is associated with child phone and tablet use |
| Capulong et al. 2025;^63^ Philippines; cross-sectional | N= 124  Age: 28.76 (8.81) months  Sex: 47.6% male  SES:  91.6% of the mothers held undergraduate and graduate degrees (or with some graduate units);  80.7 % were employed with the highest percentage of occupation under the professional or technical workers category based on the Philippine Standard Industrial Classification, e.g., doctor, lawyer, teacher (PSA, 2009). | Type and duration:  Tablets and smartphones  Weekday time: 1 hour and 44 minutes (106.95); range 0 minute to 11 hours  Weekend time: 1 hour and 53 minutes (113.84), range 0 minute to 11 hours.  Overall screen time on a typical day: 1 hour and 46 minutes (101.38), range from 4 minutes to 11 hours.  Content:  Watch programs: 96%  View photos: 72.6%  Video chat/call: 59.7%  Listen to music: 47.6%  Play apps: 44.4%  Read (being read to) e-books: 10.6%  Reporting type: touchscreen questionnaire constructed for this study based on a range of other studies | *Individual (smartphone and tablet):*  Child age  *Interpersonal (smartphone and tablet):*  Parent seeing as a benefit for the child; Parents seeing as a benefit for parents; Parental mediation strategies (Restrictive Strategy (set rules); Active Strategy (explain media content to the child); Co-use Strategy (use screen with the child); Technical safety (technology-supported safety measures such as apps designed to protect children’s online safety); Diversionary (parents actively and intentionally divert their children by encouraging them to engage in alternative, off-screen activities) | Child age, parent’s active and diversionary mediation  strategies, and the perceived maternal benefits of children’s touchscreen predict screen  time. |
| Chaibal et al. 2022;^56^ Thailand; cross-sectional | N= 85  Age: 4.05 (0.91) years  Sex: 50.59% male  SES:  Education Father:  Primary school: 37.5% (n=30); Secondary school: 43.75% (n= 35); Vocational certificate/Bachelor’s/Master’s and Doctorate: 18.75% (n= 15)  Education Mother:  Primary school: 20.25% (n=16); Secondary school: 59.50% (n= 47); Vocational certificate/Bachelor’s/Master’s and Doctorate: 20.25% (n= 16)  Family income:  <15,000 baht: 68.24% (n=58); ≥15,000 baht: 31.76% (n=22) | Type and duration:  Child use smartphone and tablet: 82.78 (62.82) min/day  Content: most children  spent time watching cartoons in the  Thai and English languages.  Reporting type: parent-reported questionnaire questions included: time since start of smartphone and tablet use, media type, time of day of device  use and smartphone usage duration over seven consecutive days | *Individual (smartphone and tablet):*  Age at start of tablet use  Chronological age  *Interpersonal (smartphone and tablet):*  Duration spent on tablet use by father; Duration spent on tablet use by mother; Duration spent on tablet use by relatives | Children's smartphone and tablet usage duration showed a significant positive correlation with the time mothers and relatives spent on smartphones and tablets. |
| Chakranon et al. 2026;^53^  China; longitudinal | N= 590  Age: 3.6 (0.2) years  Sex: 50.68% male  SES:  Family monthly income:  <=NTD 100000 = 59.62%;  >NTD 100000 = 40.38%  Parental education:  Both parents with a graduate school degree or higher: 55.29%;  Either parent with a graduate school degree or higher: 28.33%;  Both parents with a college degree or lower: 16.38% | Type and duration: Smartphone and tablet    Smart device use (hours/day):  At age 3: 0.57 (0.78)  At age 4: 0.55 (0.89)  At age 5: 0.52 (0.77)  Content: not reported  Report type: Parent-reported questionnaire, not validated but used in previous studies | *Individual (smartphone and tablet):*  Children’s outdoor activity  *Interpersonal (smartphone and tablet):*  Maternal age; Maternal BMI; Maternal depression;  Paternal BMI; Parental educational level; Family monthly income; Parity;  Gestational age | There was a bidirectional associations between increased smart device use and increased BMI, which in increase smart device use among young children, particularly among boys and children with a low level of mother-child interactions. |
| Chen et al. 2020;^32^  China; cross-sectional | N= 4,907  Age: 49.64 months (3-6 years)  Sex: 52.9% male  SES: not reported | Type and duration:  Child using tablets and smartphones: 1.102 (1.097) hours  Content: not reported  Reporting type: parent reported survey indicating hours child use tablet and smartphone | *Individual (smartphone and tablet):*  Sex; Age; TV use  Computer use; frequency child self-read  *Interpersonal (smartphone and tablet):*  Efficacy in parenting style; Income; Single parent; Parental Education; Frequency Parent read child  *Environment (smartphone and tablet):*  Number of books | High tablet/smartphone users more likely to be lower income, lower parental education and lower parental efficacy |
| Danet et al., 2022;^40^  USA; cross-sectional data extracted from longitudinal study | N= 368  Age: 3.82 (0.53) years  Sex: 51.6% male  SES:  Education  6.8% less than High school/GED, 63.3% had a college degree. 27.7%  Household income:  Income-to-needs ration: 3.00 (1.71) | Type and duration:  Child use of mobile device ≥1 h/day = 51.4%  Content:  Use for Educational apps 39.7%; Use age inappropriate apps 29.6%, Use streaming video apps 29.6%.  Reporting type: passive sensing app (Chronicle) or sent screenshots of app usage if used iOS^82^ | *Individual (smartphone and tablet):*  Inhibitory self-control index; Flexibility index; Emergent meta cognition Index; General Executive Composite | Children with weaker overall executive functioning use mobile devices |
| Gago-Galvagno et al., 2023;^52^  Argentina; cross-sectional | N= 114  Age: 27.48 (7.31) months  Sex: 52% male  SES:  Socioeconomic status:  56% low socioeconomic status | Type and duration:  Child cell phone use: 32.93 (62.21) min  Child tablet use: 1.34 (6.22) min  Content: not reported  Reporting type: parent questionnaire reporting device type, and duration (min per day) | *Individual (smartphone and tablet separate):*  Child PC use; Child book use; Child TV use  *Interpersonal (smartphone and tablet separate):*  Mother education; Father education; Mother occupation; Father occupation; Background TV; Share TV with adult; Share cell phone with adult; Share PC with adult; Share tablet with adult; Share internet with adult | Higher parent’s education and occupation were associated with increased  time tablet use, |
| Gago-Galvagno et al., 2024;^31^  Argentina; cross-sectional | N= 33  Age: 27.2 (0.4) months  Sex: 49% male  SES:  28 families from low-to-medium-SES attending daycare centres  4 families from homes in shantytowns of the Autonomous City and the Province of Buenos Aires | Type and duration:  Smartphone and tablet  Smartphone: 1.303 (1.468) hours  Tablet: 0.030(0.174) hours  Content:  The types of content across all devices (tablet, smartphone, TV and PC): entertainment (n = 13, 39.4%), educational (n = 10, 3.3%), and music (n = 10, 3.3%).  Content appropriate for their age (n = 25, 75.8%);  Content for adults and/or older children (n = 8, 24.2%).  Reporting type:  Parent-reported screen use (non-validated) | *Interpersonal (smartphone and tablet separate):*  Background TV | There is a need to increase quality interactions between parent and child |
| Gou et al 2018;^67^  China and Australia; cross-sectional | N= 1,171  Age: 3 to 7 years  Sex: not reported  SES: not reported | Type and duration:  Child tablet use: 31 min weekdays and 46 min weekends. 20.6% of sample was used on weekday and 28.5% use on the weekends  Child smartphone use: 22 min weekday and 25 min weekends. 41% of sample was used on weekday and 50.3% use on the weekends.  Content: Smartphones: Watching cartoon or kids program 79.1%; watching programs not for kids 1.5%; Educational apps 28.5%; Arts apps 16.6%; Playing games 38.2%, Making phone call and video chat 41.5%, Others 5.1% Tablet: Watching cartoon or kids program 87.2%; watching programs not for kids 1.5%; Educational apps 44.4%; Arts apps 16.1%; Playing games 47.9%, Making phone call and video chat 11.7%, Others 2.4%  Reporting type: questionnaires completed by child primary caregiver | *Individual (smartphone and tablet separate):*  Age; Sex  *Interpersonal (smartphone and tablet separate):*  Mother education; Father education; Family income; Sibling presence; Device use caregiver | Comparative study between China and Australia, which looked at use and parents’ perceptions of mobile device. No direct conclusions about the correlates of mobile device use. |
| Howie et al 2020; ^68^  Australia and USA; cross-sectional | N= 96  Age: 5.5 to 64.3 months  Sex: 51.5% male  SES:  Education: 47.9% postgraduate degree | Type and duration:  Child tablet use: weekday: 19.5 (SD 60.3) min/day; weekend: 23.8 (SD 46.0) min/day  Child smartphone use: Weekday: 6.4 (SD 19.4) min/day; Weekend: 8.2 (SD 24.0)  Content: Tablet: 44% educational, 34% videos; 6% Games; 5% Social and 11% General Mobile phones: 50% videos, 25% Educational, 7% games, 3% Social and 15% general  Reporting type: Technology Use Questionnaire (TechU-Q(parent) – parent proxy report of young children’s (birth to 5 years), which was validated within the study. | *Interpersonal (smartphone and tablet separate):*  Parental mobile use; Parental mobile use at weekends; Parent mobile use at weekdays | Parent use of mobile devices were associated with their child’s use. |
| Jamil et al., 2021;^55^  Malysia; cross-sectional | N= 463  Age: 4 to 6 years  Sex: 50.1% male  SES:  Education  Spouse education level: Primary 5%; Secondary 70%, 25%Tietary  Working status:  Working status: 61% Yes; 39% No | Type and duration:  Tablet and smartphone use: 71% (n=313)  Content: not reported  Reporting type: mobile device usage was adapted from the pre-school technology survey  questions developed by McCloskey et al.^72^ Mobile device usage refers to the time duration a pre-schooler uses the device in a day, but the study divided mobile device users into two categories, namely non-regular users (using the device ≤ 60 min/day)  and regular users (using the device > 60 min/day). | *Individual (smartphone and tablet):*  Child age; Presence of siblings; Sex; Number of siblings; Birth order  *Interpersonal (smartphone and tablet):*  Age parent; Medical problem child; Ethnicity; Post natal complications; Marriage status; Level of education; Working; Income; Childcare (family member vs. non-family member); Spouse age (years); Spouse level of education; Spent time with children; Family type (nucleus vs extended); Medical problem parent | Parental and family factors affect the high usage of mobile devices among pre-school children. |
| Kara et al., 2025; ^48^Turkey; cross-sectional | N= 109  Age: 43 (39%) aged 3-4 years old and 66 (61%) aged 5-6 years  Sex: 64.2% boys  SES: not reported | Type and duration:  Smartphone and tablet:  Weekly 8 hours $\leq$:52.3% (n=57)  Weekly 8 hours > : 47.7% (n=52)  Content: not reported  Reporting type: Parent-reported questionnaire (non-validated) | *Interpersonal (smartphone and tablet):*  Parent Attitude Scale: Democratic; Authoritarian; Tolerant  Internet Parenting Style Scale: Parental control; Parental warmth | Parental control, parental warmth and democratic attitudes were higher in children with no with mobile device use. |
| Kardas et al., 2024;^45^  Turkey; cross-sectional | N=858  Age: 66 months  Sex: 52.8% male  SES:  Income per month:  Below the national poverty threshold (<1500 tl): 4.6% Above the national poverty threshold (1500-4500 tl): 46.4% Middle income (4500-10000 tl): 43.5% High income (>10000 tl): 5.6% | Type and duration:  Time spent on smartphone: Not use: 37.4%; ≤30 min: 22.9%; >30 - ≤120 min: 33.7%; >120 - ≤180 min: 3.2%; >180 min: 2.5%  Content: Most popular choice on smartphone: Games: 47.8% Taking photos: 12.9% Music: 4.3% Internet surfing: 9.7% Else: 25.3%  Reporting type: parent reported questionnaire covering the age children were introduced to media devices, children’s daily screen time, and the media habits of the parents. | *Individual (smartphone):*  BMI  *Interpersonal (smartphone):*  Parental TV time; Parental internet time; Mothers age; Fathers age | Parents’ education levels, parents’ screen time and were related to children’s media habits |
| Kim et al., 2021;^33^  Korea; cross-sectional | N=353  Age: 2 to 5 years  Sex: 51.7% male  SES:  Education:  Paternal education: High school or below: 12.5% (n=50); College or above 87.5% (n=353)  Maternal education: High school or below: 12.5% (n=50); College or above 87.5% (n=350)  Monthly household income: <W4,000,000 = 50.5% (n=202); W4,000,000 = 49.5% (n=198) | Type and duration:  Time and content not reported  Content: not reported  Reporting type: Parent reported type of device,  frequency and average amount of time spent using smart devices during the past month. The responses were divided as weekday or weekend. Parents also reported the  children’s age at first smartphone usage. | *Interpersonal (smartphone only):*  Adult smartphone addiction; Maternal employment status; Parenting stress; Parent depression; Parent anxiety | Parents smartphone addiction can affect early smartphone exposure on children. |
| Kristo et al., 2021;^35^  Turkey; cross-sectional | N= 104  Age: 2 to 5 years  Sex: 60.6% male  SES:  Education:  Mothers’ education:  1% (n = 1) illiterate; 1.9% (n = 2) primary school graduate.  4.8% (n = 5) middle school graduate; 40.4% (n= 42) high school graduate; 45.2% (n = 47) university graduate; and 6.7% (n = 7) postgraduate level.  Fathers’ education: 1% (n = 1) illiterate; 3.8% (n = 4) primary school graduate; 11.5% (n = 12) middle school graduate; 32.7% (n = 34) high school graduate; 42.3% (n = 44) university graduate; and 8.7% (n = 9) postgraduate level. | Type and duration:  Tablet and smartphone use: tablet/smartphone 0–1 h/day, 23.1 % (n = 24); use 1–2 h/day, and 7.7 % (n = 8) use 2+ h/day tablet/smartphones,  21.2% (n = 22) of children do not use tablet/smartphones.  Content: not reported  Reporting type: validated parent reported questionnaire^78^ | *Individual (smartphone and tablet):*  Weight status  Nutritional score | There is no association between tablet/smartphone use duration and children’s weight status. However, tablet/smartphone use and were significantly associated with children’s nutrition score. |
| Kumruangrit et al. 2022;^57^ Thailand; cross-sectional | N= 1,100  Age: 3.5 (range 2 to 5) years  Sex: proportion of girls and boys are similar  SES:  Education:  Completed elementary education (33.7%). Graduated from high school (52.1%) bachelor’s degrees (10.3%) | Type and duration:  Smartphone and tablet use: Monday to Sunday: 1h 33 min (1h10 min); Monday-Friday: 1h25 min (1h 04 min); Saturday and Sunday: 1h 57 min (1h52 min)  Content: School-related (language training videos): 8.8%; Listening to videos (2.7%); Watching media for children: story -telling (55.2%); Watching cartoons/movies (55.2%); Children's television shows via YouTube or TikTok (23.3%); Playing games (2.7%)  Reporting type: parent- questionnaires who were internally validated by experts and piloted with 30 parents, but validation data not provided. | *Interpersonal (smartphone and tablet):*  Frequency of primary parent smartphone use; Frequency of primary parent smartphone use while staying with children; Frequency of family members smartphone use while staying with children | Children mobile devices use was higher on families who enable than to use it. |
| Lauricella et al., 2015;^41^  USA; cross-sectional | N= 2,225  Age: 4.2 (2.58) years  Sex: 50% male  SES:  Household income:  Average household income was between $40,000 and $59,999, and median income was between $60,000 and $74,999 | Type and duration:  Tablet use: 29 (43) min  Smartphone use: 13 (32) min  Content: not reported  Reporting type: Questions for screen time were based on Common Sense Media (2011) national survey. It included type of devices in the household and amount of time the child spent using the device on a typical  weekday and weekend day | *Individual (smartphone and tablet separate):*  Child age  *Interpersonal (smartphone and tablet separate):*  Parental screen time; Parent attitudes | Results from linear regression analyses indicate that parents' own screen time and parents attitudes were strongly associated with child screen time. |
| Lee et al., 2013;^37^  South Korea; cross-sectional | N= 500  Age: 0 to 6 years  Sex: 53.7% male  SES:  Education  17.3% high school or lower; 24.5% college; 47.8% University graduate; 10.4% graduate school.  Monthly income on average:  36.4% earned 2-3 million KRW; 36.6% earned 3-4 million KRW; 27% earned 4-5 million. | Type and duration:  Smartphone use  Average all children: 1 hour 92 children (64.3%), 2 hours 34 children (23.8), 17 children (11.9%).  Content: children use smartphone mainly to watch cartoon videos and listening to stories and songs. Education application in the aspect of learning was relatively low.  Reporting type: customised questionnaire | *Individual (smartphone only):* Sex; Age  *Interpersonal (smartphone only):*  Mother’s child rearing efficacy. | Parental efficacy of  mother is positively associated with the number of use and  average use hours of children. |
| Lee et al., 2022;^34^  South Korea; cross-sectional | N= 283  Age: 4.3 (SD = 0.9) years  Sex: 45.9% male  SES:  Household monthly income (Korean 10 thousands won): <200: 0.4%; 201–300: 10.3 %; 301–400: 17.3 %; 401–500: 20.1 %; 501<: 51.9 % | Type and duration:  Smartphone use: 1.1 (1.5) hours/daily  Content: not reported  Reporting type: questionnaire focusing on preschooler usage time.  The National Information  Society Agency questionnaire for smartphone^71^ overdependence for children (ages 3–9 years old). | *Interpersonal (PSU):*  Mothers' emotional intelligence; Mothers’ negative parenting behaviour; Mother-child attachment instability; Mother's smart device usage | Attachment instability of preschoolers has a strong relationship with their  smart device overdependence. |
| Lee et al., 2023;^35^  South Korea; cross-sectional | N=357  Age: 1 to 3 years - 46.5% 4-6 years - 53.5%  Sex: 47.6% male  SES:  Household income: Less than USD 1,800: 2.8%; USD 1,800–2,700: 10.9%; USD 2,700–3,600: 25.5%; USD 3,600–4,500: 22.7 %; USD 4,500–5,400: 16.5%; More than USD 5,400: 21.6% | Type and duration:  Smartphone use: Weekdays: Less than 10 min: 9.8%; 10–20 min: 14%; 20–30 min: 19.0 %; 30–40 min: 17.1 %; 40–60 min: 18.5 %; 60 –90 min: 12.9 %; More than 90 min: 12.9%  Weekends: Less than 10 min: 7.3%; 10–20 min: 10.4%; 20–30 min: 15.7 %; 30–40 min: 16.0 %; 40–60 min: 17.4 %; 60 –90 min: 16.0 % ; More than 90 min: 17.4%  Content: not reported  Reporting type: parent reported questionnaire | *Interpersonal (PSU):*  Parent smartphone overuse; Parenting stress;  Parenting efficacy | Low parenting efficacy and high parenting stress increase smartphone overuse children. Parents’ smartphone overuse also influences children’s smartphone overuse. |
| Lee et al., 2024;^38^  South Korea; longitudinal | N= 313  Age: 4.5 (0.82) years  Sex: 49.8% male  SES: Monthly family income  Less than $765 : 2 (0.6%)  $765–$1,530: 9 (2.9%)  $1,530–$3,060: 87 (27.8%)  $3,060–$4,590: 130 (41.5 %)  $4,590 or more: 85 (27.2%) | Type and duration:  Problematic Smartphone Use  Weekdays  No use: 127 (40.6%)  Less than 1 hr: 113 (36.1%)  1–2 hr: 61 (19.5%)  2–3 hr: 11 (3.5%)  3–4 hr: 1 (0.3%)  4 hr or more: 0 (0.0%)  Weekend  No use: 109 (34.8%)  Less than 1 hr: 95 (30.4%)  1–2 hr: 79 (25.2%)  2–3 hr: 24 (7.7%)  3–4 hr: 6 (1.9%)  4 hr or more: 0 (0.0%)  Content: not reported  Reporting type:  Child Smartphone Addiction Observer Scale^1^ | *Individual (PSU):*  Sex; Age; Smartphone use frequency; Smartphone use duration; Age at first smartphone use  *Interpersonal, (PSU):*  Parent’s smartphone addiction proneness; Parental control over children’s smartphone  use; Income | Lack of control over children’s smartphone use, and parents’ higher smartphone addiction proneness predicted higher smartphone addiction tendency in young children. |
| Maatta et al., 2017;^60^  Finland; cross-sectional | N= 771  Age: 4.73 (0.89) years  Sex: 51% male  SES:  Education  Maternal education: low = 30%; medium = 41%; high = 29%  Paternal education: low = 45%; medium = 33%; high = 22%  Household income:  low = 32%, medium = 34%, high = 34% | Type and duration:  Tablet/smartphone use: 21.82 (26.18) min  Content: not reported  Reporting type:  Diary where parents reported daily use of tablet and smartphone, including frequency and hours/ minutes in total. Diary has been based on previously validated method.^77^ | *Interpersonal (tablet and smartphone):*  Maternal education; Paternal education; Household income | Children of middle household income family had a lower risk of using tablets and smartphones compared with their counterparts with a high household income. |
| Martín-Aragoneses et al., 2025;^65^ Spain; cross-sectional | N=207  Age: 56.22 (9.76) months  Sex: 43.5% male  SES:  With financial difficulties: n= 4 (1.9%)  Economic capacity to meet essential needs: n= 19 (9.2%)  Economic capacity to save some money: n= 166 (80.2%)  High income, no financial worries: n= 18 (8.7%) | Type and duration:  Smartphone use: 19.88 (40.95) min  Content: not reported  Reporting type: Parent-self reported questionnaire (non-validated) | *Individual (smartphone):*  Age  *Interpersonal (smartphone):*  Percentage of time the child spends alone on smartphone | Percentage of time the child spend alone on the smartphone is positively associated with smartphone use. |
| McDaniel et al., 2020;^42^  USA; longitudinal | N= 366 (baseline)  N= 337 (follow-up)  Age: 3.0 (1.2) years  Sex: 45% male  SES:  Education:  72% parents had at least bachelor’s degree  Income:  Median yearly household income $69,500 mean yearly household income $74,870 | Type and duration:  Daily use of tablet: never: 55.3%; 1-15 mins 19.4%;16-30 mins 12.4%; 31-60 mins 7.1%; 1-2 hours 3.5%; >2 hours 2.4%    Daily use of smartphone: never: 72%; 1-15 mins: 26%; 16-30 mins: 9% 31-60 mins 7%; 1-2 hours 3%; >2 hours 2%  Content: not reported  Reporting type: parent report on the time the child spends on the device | *Interpersonal (tablet and smartphone separate):*  Parenting stress | We found  that child behavioural difficulties predicted greater media use  overall—and more TV, game, and tablet use specifically—a  relationship that was mediated by higher parenting stress. |
| Nikken et al. 2017;^62^  Netherland; cross-sectional | N=1,381  Age: 3.67 (1.83) years  Sex (1= male): 0.51 (0.5)  SES  Education:  11% of the parents ‘very low’ educational level (no education/only primary education); 34% ‘low’ level (vocational secondary education); 38% ‘high’ educational level (university preparatory education, bachelor’s degree); 17%‘very high’ level (university education/master’s degree).  Annual family income:  5% annual family income up to 40,000 Euro; 54% annual family income between 40,000 and 67,000 Euro per  year; 21% family income of more than 67,000 Euro per year. | Type and duration:  Time spent on touchscreen devices: 52 min (reported from Figure 1)  Content: not reported  Reporting type: online questionnaire to parents, which asked parents how many hours and or minutes on a regular day their child spend on electronic devices at home | *Interpersonal (tablet and smartphone):*  Parental touchscreen use | Parent’s touchscreen use is associated with their children. |
| Park et al., 2018;^36^  South Korea; cross-sectional analysis of a cohort study | N= 400 (baseline)  N= 380 (follow-up)  Age: range 2 to 5 years; 2 (15.3%), 3 (43.9%), 4 (30.5%), 5 (10.3%).  Sex: 52.1% male  SES  Education: 88.7% mothers had a college education.  Monthly household income: 50% households < KRW 4,000,000 (low income). | Type and duration:  Overuse (≥ 1 hr /day):  Smartphone: n= 38 (10%)  Tablet: n= 24 (6.3%)  Content: not reported  Reporting type: customised questionnaire which questioned the type of media device and average amount of screen time that children spent on each type of media device during the past month | *Individual (tablet and smartphone separate)*:  Sex; Age  *Interpersonal (tablet and smartphone separate):*  Main caregiver (parent vs. grandparents); Paternal education; Maternal education; Maternal employment status; Monthly house income; Maternal depression | Maternal depression was not associated with smartphones and tablet use |
| Park et al., 2021;^21^  South Korea; cross-sectional | N=1,378 (with problematic smartphone use (PSU) n= 236; without PSU n= 1,142.  Age: PSU: 4.8 (1.06) years non-PSU: 4.6 (1.11) years  Sex: non-PSU - 46.1 % male; PSU - 42.4 % male  SES:  Household income:  PSU: less than 2000 KRW: 2.5%; 2000-4000 KRW: 53.8%; 4000-6000 KRW: 32.2%; 6000-8000 KRW: 5.1%; 8000-10000 KRW: 3.8%; 10000+ KRW: 2.5%  Non-PSU: less than 2000 KRW: 3.0%; 2000-4000 KRW: 36.7%; 4000-6000 KRW: 23.7%; 6000-8000 KRW: 13.0%; 8000-10000 KRW: 8.1%; 10000+ KRW: 15.5% | Type and duration:  Hours of day per use  PSU: Average 2.5 (1.86) hours per day  non-PSU: Average 0.8 (0.47) hours per day  Content:  Degree of use  PSU: Education: 3.0 (2.62); Web surfing - 0.9 (1.87); Games - 4.2 (2.29); TV/Video - 4.0 (2.30); Music - 2.7 (2.50); Webtoons/Fiction - 1.1 (2.19); Messenger - 1.4 (2.27); Social networking services - 0.6 (1.44)  Non-PSU: Education - 3.1 (2.01); Web surfing - 1.4 (1.98); Games - 3.1 (2.38); TV/Video - 2.6 (2.54); Music - 2.6 (2.46); Webtoons/Fiction - 0.6 (1.56); Messenger - 1.4 (2.09); Social networking services - 0.7 (1.65)  Reporting type: Problematic smartphone use for preschool children was  measured with the Korean-language Smartphone Overdependence Scale (S-scale) for children^71^ | *Individual (PSU):*  Sex; Age  *Interpersonal, (PSU):*  Family size (number of members); Parent's employment status; Household income; Number of preschool children in the household; Main caregiver (ref=grandparent); Main caregiver's age; Main caregiver's perception | Children who use smartphones to watch TV shows or videos for entertainment had a significantly higher odds of PSU. On the other hand, there was no significant association between PSU risk and use smartphones for education, games, and social networking. |
| Pempek & McDaniel 2016;^43^  USA; cross-sectional | N= 358  Age: 2.21 (0.89) years  Sex: not reported  SES  Education: 72% had a college degree  Income: Mean income was U$ 64,039 (42,702). | Type and duration:  All sample use of tablet:  Never: 249 (69.9%);  1–15 min 45 (12.6%);  16–30 min 30 (8.4%);  31–60 min 18 (5.1%);  >60 min 14 (3.9%)  Content: not reported  Reporting type: Parents reported their child’s frequency of tablet use (i.e., using an iPad, Kindle, or other tablet) on a typical day on a 10-point scale: 0 (Never), 1 (1–15 min),  2 (16–30 min), 3 (31 min to 1 h), and so on to 9 (7 or more hours). App content was reported via Common Sense Media survey.^81^ | *Individual (tablet):*  Child age  *Interpersonal (tablet):*  Mothers tablet use; Mothers personal wellbeing (e.g., depression, role overload); Mothers relational wellbeing (e.g., relationship satisfaction, coparenting quality, relational conflict). | For families who  owned a tablet, child’s frequency of use was positively associated with child’s age and mother’s use and negatively associated with mother’s relational well-being. |
| Rathnasiri et al. 2022;^66^  Sri Lanka; cross-sectional | N= 340  Age: 3 to 5 years  Sex: 49/1% male  SES:  Maternal education:  Primary education 0.3%; Secondary education (Ordinary Level) 15.4%; Secondary education (Advance Level) 47.3% ; Higher education 36.9%  Income: Monthly family income <LKR 25,000 5.7%; LKR 25,000–50,000 38.9%; LKR 50,000–100,000 38.3%; >LKR 100,000 17.2% | Type and duration:  Tablet screen time per day: none 92.6%; <30 min 4.4%; 31min-1h 2.3%; 1-2h 0.3% 2-3h 0.3%  Smartphone screen time per day: none 38.6%; <30 min 41.3%; 31min-1h 12.7%; 1-2h 3.5%; 2-3h 3.6%; 3-4h 0.3%  Content: not reported  Reporting type: Customised questionnaire in which parent reported the average time the child spent per day for each device. | *Individual (smartphone):*  Sex; Age  *Interpersonal (smartphone):* Mother educational level, Father educational level, Mother employment status; Number of children; Monthly family income | Higher paternal education, maternal employment and being the only child were significantly associated with electronic device use. |
| Rocha et al. 2023;^51^  Portugal; cross-sectional | N= 340  Age: 18 to 57 months  Sex: 54.1% male  SES:  Education level: 34.1% lower school degree 65.9% university degree | Type and duration:  Tablets and smartphones: 42.48 (52.52) min/day  Content: not reported  Reporting type: Questionnaire adapted from Nikken et al^70^ with questions related to the frequency of electronic device  use by the child (type of device and screen time) | *Individual (tablet and smartphones):*  Paediatric symptoms; Watch TV, TV time, Gender  *Interpersonal (tablet and smartphones):*  Parent academic level; Parent working hours; Parent university degree. | Paediatric symptoms and parents’ working hours appeared as the predictor  to multimedia device usage. Likewise, the results indicated that a higher mean of multimedia device usage in children of parents without an academic degree. |
| Rodrigues et al. 2020;^49^ Portugal; cross-sectional | N= 1,860  Age: 4.47 (0.67) years  Sex: 52.6% male  SES:  Low SES - 22.6%; Medium SES - 38.6%; High SES - 38.8% | Type and duration:  Figure 2 reported smartphone time range across 7 day: Males: 12 min/day to 28 min/day; Females: 20 min/day to 32 min/day  Tablet time range across 7 day: Males: 20 min/day to 40 min/day; Females: 20 min/day to 30 min/day  Content: not reported  Reporting type: previously used parental questionnaires.^80^ Parents were asked to report the average time per day that the child spent on the tablet or a smartphone. Separate responses were collected for  weekend and weekday. Response options were none = 0, less than 1 h per day; 1 h/d = 60 min, 2 h/d = 120 min, 3 h/d =180 min, 4 h/d = 240 min, and more than 4 h/d. | *Interpersonal (smartphone and tablet separate):* Socioeconomic position | Socioeconomic position explained some of the variance in children’s screen time, with screen time being higher in children from socioeconomic disadvantaged backgrounds. |
| Rodrigues et al., 2022;^50^Portugal; cross-sectional | N= 6,347 in total age range 3 to 10 years old.  N= 1,863 children aged 3 to 5 years old  Age: all sample 3 to 10 years, but separate analysis for 3 to 5 years  Sex: 52.2% male  SES:  Education:  Father's Education 3- to 5-year-old group:  Low: Boys: 21.0% (196) Girls: 20.0% (172);  Medium: Boys: 38.8% (362) Girls: 38.5% (331);  High: Boys: 40.2% (375) Girls: 41.5% (357) | Type and duration:  Screen time tablet and smartphone not reported  Content: not reported  Reporting type: Reporting type: previously used parental questionnaires.^80^ Parents were asked to report the average time per day that the child spent on the tablet or a smartphone. Separate responses were collected for weekend and weekday. Response options  ranged from “none” to more than 4 hours per day for all questions. | *Environmental (smartphone and tablet separate):*  Good environment for physical activity; Good environment for walking; High crime rate | Social environment, compared with the  Physical environment, is more relevant for children’s screen time, particularly  among younger children. Neighbourhood features were more strongly  correlated with girls’ screen time than boys’. |
| Sari et al. 2021;^46^  Turkey, cross-sectional | N= 210  Age: 3 to 6 years  Sex: 49% male  SES:  Maternal education:  Primary school -3.4%; Secondary school - 4.8%; High school - 29.8%; University - 52.9%; Master’s degree - 9.1%  Paternal education:  Primary school -0.5%; Secondary school - 2.9%; High school - 3.9%; University - 59.3%; Master’s degree - 10.3% | Type and duration:  Reported daily hours of using a smartphone and daily hours of using a tablet/internet but unclear how to extract from tables.  Content: not reported  Reporting type: Parents responded a questionnaire in which they said how long they spend on tablets and smartphones | *Individual (smartphone and tablet separate):*  Child temperamental traits: activity level; anger/frustration; approach; attentional focusing; discomfort; falling reactivity and soothability; fear; high intensity pleasure; impulsivity; inhibitory control; low intensity pleasure; perceptual sensitivity; sadness; shyness; smiling and laughter)  *Interpersonal (smartphone and tablet separate):*  Parental attitudes:  excessive motherhood ; democratic attitude and recognition of equality; hostile and rejective attitude; discord between parents; authoritarian attitude | An association was found between poor parenting attitudes and smartphone and tablet/internet usage durations.  A relationship was found between poor parenting attitudes and smartphone and tablet/internet usage durations. Authoritarian  parents let their children use the internet/tablet  and smartphone for more hours.  It was also noted that  the daily smartphone usage shortened as soothability increased and this relationship was affected by authoritarian control |
| Shawcroft et al. 2023;^39^  USA; longitudinal | N= 519 (baseline)  N= 435 (follow-up)  Age: 29.68 (3.73) months  Sex: 52.87% male  SES:  Annual income  7.36% $15,000;  6.90% $15,000–$24,999;  7.36% $25,000–$34,999;  12.18% $35–$49,999;  21.38% $50,000–$74,999; 16.32% $75,000–$99,999; 19.31% $100,000 -$149,999; 8.97% $150,000 or more | Type and duration:  Problematic Smartphone Use at baseline: 1.71  Did not report time spent on tablet  Content: not reported  Reporting type:  Time spent on tablet reported via customised questionnaire  PSU: nine-item Problematic Media Use Measure–short form (PMUM-SF).^69^ | Analysis contained both PSU and time spent on tablet  *Interpersonal (tablet):* Ethnicity, Income, No tablet rules; Ambiguous tablet rules  *Interpersonal (PSU):*  Ethnicity, Income, No tablet rules; Ambiguous tablet rules | Parents have more rules around child TV use than tablet use. Children who were not allowed to use tablets at all displayed lower  levels of problematic media use 2 years late |
| Thompson et al. 2026; ^44^  USA; cross-sectional | N= 313  Age : 21.2 (3.0) months  Sex: 48.6% male  SES: Employed: 41.5%  Education in years: 11.6 | Type and duration:  Tablet and smartphone:  27.9 (6.4 to 66.4) min per day  Content: not reported  Reporting type:  7-day daily screen use diary tracking their toddler’s mobile device use (smartphones and tablets) in 15-minute increments from 5 AM  to midnight. Validated questionnaire similar to other studies.^2^ | *Individual (tablet and smartphones):* age; gender; BMI-score  *Interpersonal (tablet and smartphones):*  1) mother duration mobile use  Screen-related parenting practices:  Restrict time (mobile device);Co-view (mobile device);Behaviour regulation ( mobile device); Screen restrict content (mobile device) | The duration of maternal screen use and screen-related parenting practices, should be considered when promoting healthy screen use in toddlers in Mexican American families. |
| Yang et al. 2022;^64^  Singapore; cross-sectional | N=154  Age: 61.42 (8.93) months  Sex: 51.9% male  SES:  College graduates: 48.1% | Problematic smartphone use average not reported  Content: not reported  Problematic smartphone use was measured by the  Adapted version of smartphone addiction scale (short version),^73^ which was originally developed for adolescents. Some items not specific for young child were excluded. Mothers were instructed to rate their  child’s behaviours on a 6-point Likert scale (1 = strongly disagree; 6 = strongly agree) | *Individual (PSU):*  Age, Sex  *Interpersonal (PSU):*  Mother's ethnicity; Mother's marital status; Mother's age; Mother's education; Restrictive mediation; Inconsistent mediation; Psychological aggression; Physical assault; Nonviolent discipline | Negative mother–child conflict resolution tactics (e.g., psychological aggression and physical assault) moderate the relationship between inconsistent maternal media mediation and young children’s problematic smartphone use  during early childhood. However, restrictive mediation interacted with neither positive nor negative mother–child conflict resolution tactics in explaining children’s PSU. |

References

1 Kim DI, Chung YJ, Lee YH, et al. Development and validation of child smartphone addiction observer scale. Korean Journal of Counseling 2015;16:369–83.

2 Mendoza JA, McLeod J, Chen T, et al. Convergent validity of preschool children's television viewing measures among low-income Latino families: a cross-sectional study. Childhood obesity 2013;9:29–34.
