# Supplementary material for: Correlates of mobile device use in young children: a systematic review and meta-analysis
Source: BMJ Public Health. 2026 Jun 17;4(2):e004305. doi: 10.1136/bmjph-2025-004305 (PMC13289221; doi:10.1136/bmjph-2025-004305)
Supplement: online supplemental file 6 [file bmjph-4-2-s006.docx]

**Supplementary File 6**

**Quality Assessment**

Table 1. Quality Assessment of Cross-sectional Studies

| Reference | 1 | 2 | 3 | 4 | 5 | 6 | 7 | 8 | 9 | 10 | 11 |
| --- | --- | --- | --- | --- | --- | --- | --- | --- | --- | --- | --- |
| Abdullah 2022^54^ | Yes | Yes | Can't tell | Yes | Can't tell | Yes | Yes | Yes | Yes | No | Yes |
| Abdulla 2023^59^ | Can't tell | Yes | No | Can't tell | Can't tell | Yes | Yes | Yes | Yes | Can't tell | Yes |
| Ali 2022^61^ | Yes | Yes | No | Yes | Yes | Yes | Yes | Yes | Yes | Can't tell | Yes |
| Bui 2022^5658^ | Yes | Yes | Can't tell | Can't tell | Can't tell | Can't tell | Yes | Yes | Yes | No | Yes |
| Chaibal 2022^56^ | Yes | Yes | Can't tell | No | Yes | Yes | Yes | Yes | Yes | Can't tell | Yes |
| Chen 2020^32^ | Yes | Yes | No | Yes | Yes | Yes | Yes | Yes | Yes | Can't tell | Yes |
| Capulong 2025^63^ | Yes | Yes | Can’t Tell | Yes | Yes | Can’t tell | No | No | Yes | Yes | Yes |
| Danet 2022^40^ | Yes | Yes | Can't tell | Yes | Yes | Can't tell | Yes | Yes | Yes | Can't tell | Yes |
| Gago-Galvagno 2024^63^ | Yes | No | Yes | Yes | Yes | No | No | Yes | Yes | No | Yes |
| Gago-Galvagno 2023^52^ | Yes | Yes | Yes | No | Yes | No | Yes | Yes | Yes | Can't tell | Yes |
| Gou 2018^67^ | Yes | Yes | Yes | No | Can't tell | Yes | Yes | Yes | No | Yes | No |
| Howie 2020^68^ | Yes | Yes | No | Yes | Yes | No | Yes | Yes | Yes | Can't tell | No |
| Jamil 2021^55^ | Yes | Yes | Yes | Yes | Yes | Can't tell | No | No | Yes | Can't tell | Can't tell |
| Kara 2025^48^ | Yes | No | Can’t tell | Yes | Yes | Yes | Yes | Yes | Yes | No | Yes |
| Kardas 2023^45^ | Yes | Yes | Can't tell | No | Yes | No | Yes | Yes | Yes | Can't tell | Yes |
| Kim 2021^33^ | Yes | Yes | Can't tell | Yes | Yes | No | Yes | Yes | Yes | Can't tell | Yes |
| Kristo 2021^47^ | Yes | Yes | No | Yes | Yes | No | Yes | Yes | Yes | Can't tell | Can't tell |
| Kumruangrit 2022^57^ | No | No | Yes | Yes | No | Can't tell | No | No | No | Can't tell | No |
| Lauricella 2015^41^ | Yes | Yes | No | Yes | Yes | Can't tell | Yes | Yes | Yes | Can't tell | Yes |
| Lee 2013^37^ | Yes | Yes | No |  | Can't tell | Can't tell | Yes | No | Yes | Can't tell | No |
| Lee 2022^34^ | Yes | Yes | No | Yes | Yes | Can't tell | Yes | Yes | Yes | Can't tell | Yes |
| Lee 2023^35^ | Yes | Yes | No | Yes | Yes | Can't tell | Yes | Can't tell | Yes | Can't tell | Can't tell |
| Maatta 2017^60^ | Yes | Yes | Yes | Yes | Yes | Can't tell | Yes | Yes | Yes | Yes | Yes |
| Martín-Aragoneses 2025^65^ | Yes | No | Cant Tell | Yes | Yes | Cant tell | Yes | Yes | Yes | Yes | Yes |
| Nikken 2017^62^ | Yes | Yes | Can't tell | Can't tell | Yes | Can't tell | Yes | Can't tell | No | Yes | Yes |
| Park 2018^36^ | Yes | Yes | Yes | Yes | Yes | Can't tell |  | Yes |  | Can't tell | Can't tell |
| Park 2021^21^ | Yes | Yes | Yes | Yes | Yes | Yes | Yes | Yes | Yes | Yes | Yes |
| Pempek 2016^43^ | Yes | Yes | Yes | Can't tell | Can't tell | Can't tell | Yes | Can't tell | Yes | No | Yes |
| Rathnasiri 2022^66^ | Yes | Yes | Yes | Yes | Yes | Yes | Yes | Yes | Yes | Yes | Yes |
| Rocha 2023^51^ | Yes | Yes | Can't tell | Yes | Yes | Can't tell | Yes | Yes | Yes | Can't tell | Yes |
| Rodrigues 2020^49^ | Can't tell | Can't tell | Can't tell | Yes | Yes | Yes | Yes | Yes | Yes | Yes | Yes |
| Rodrigues 2022^50^ | Yes | Yes | Yes | Yes | Yes | Can't tell | Yes | Yes | Yes | Yes | Yes |
| Sari 2021^46^ | Can't tell | Yes | Yes | Yes | Yes | No | Yes | Yes | Yes | Can't tell | Yes |
| Thompson 2026^44^ | Yes | Yes | Yes | Yes | Yes | Yes | Yes | Yes | Yes | Yes | Yes |
| Yang 2022^64^ | Yes | Yes | Can't tell | Can't tell | Yes | Can't tell | Yes | Yes | Yes | No | Yes |
| Counts of “Yes” | 31 | 30 | 13 | 25 | 28 | 11 | 30 | 28 | 31 | 10 | 27 |

1. Did the study address a clearly focused issue? ; 2. Did the authors use an appropriate method to answer their question?; 3. Were the subjects recruited in an acceptable way?; 4. Were the measures accurately measured to reduce bias?; 5. Were the data collected in a way that addressed the research issue?; 6. Did the study have enough participants to minimise the play of chance?; 7. How are the results presented and what is the main result?; 8. Was the data analysis sufficiently rigorous?; 9. Is there a clear statement of findings?; 10. Can the results be applied to the local population?; 11. How valuable is the research?

Table 2. Quality Assessment of Longitudinal Studies

| Reference | 1 | 2 | 3 | 4 | 5a | 5b | 6a | 6b | 7 | 8 | 9 | 10 | 11 | 12 |
| --- | --- | --- | --- | --- | --- | --- | --- | --- | --- | --- | --- | --- | --- | --- |
| Chakranon 2026 | Yes | Yes | Yes | No | Yes | Yes | Can’t Tell | Yes | Yes | Yes | Yes | Yes | Yes | Yes |
| Lee 2024 | Yes | Can’t tell | Yes | Yes | Yes | Yes | Can’t Tell | Yes | Yes | Yes | Yes | Yes | Yes | Yes |
| McDaniel 2020^28^ | Yes | Yes | No | Yes | Yes | Yes | Can't tell | No | Yes | Yes | Yes | No | Yes | Yes |
| Shawcroft 2023^25^ | Yes | Can't tell | Yes | No | Yes | Yes | Can't tell | Yes | Yes | No | Yes | Yes | Yes | Yes |
| Counts of "Yes" | 4 | 2 | 3 | 2 | 4 | 4 | 0 | 3 | 4 | 3 | 4 | 3 | 4 | 4 |

1. Did the study address a clearly focused issue?; 2. Was the cohort recruited in an acceptable way?; 3. Was the exposure accurately measured to minimise bias?; 4. Was the outcome accurately measured to minimise bias?; 5. (a) Have the authors identified all important confounding factors?; 5. b) Have they taken account of the confounding factors in the design and/or analysis?; 6. a) Was the follow up of subjects complete enough? ; 6. b) Was the follow-up of subjects long enough?; 7. What are the results of this study?; 8. How precise are the results?; 9. Do you believe the results?; 10. Can the results be applied to the local population?; 11. Do the results of this study fit with other available evidence?; 12. What are the implications of this study for practice?"
