# Supplementary material for: Correlates of mobile device use in young children: a systematic review and meta-analysis
Source: BMJ Public Health. 2026 Jun 17;4(2):e004305. doi: 10.1136/bmjph-2025-004305 (PMC13289221; doi:10.1136/bmjph-2025-004305)
Supplement: online supplemental file 7 [file bmjph-4-2-s007.docx]

**Supplementary File 7**

**Summary of the correlates of problematic smartphone use, with less than three studies reporting an association.**

Table 1: Summary of individual and interpersonal correlates of problematic smartphone use, with less than three studies reporting an association.

| **Correlates** | **Problematic Smartphone use**  Number of studies (percentage, direction of association, study citation) |
| --- | --- |
| **Individual** | |
| Ethnicity | n= 1 (50% -^39^; 50% 0^64^);# |
| Smartphone use (duration) | n=1 (100% +^38^);# |
| Smartphone use (frequency) | n=1 (100% 0^38^);# |
| Age at first smartphone | n=1 (100% 0^38^);# |
| **Interpersonal** | |
| *Family characteristics and structure* | |
| Employment | Mother: n= 1 (100% +^59^); +  Father: n=1 (100% +^59^); +  Parents: n= 1(100% 0^21^); 0  Overall (mother, father, parents): n= 2 (50% +^59^; 50%0^2121, 59^); # |
| Marriage status | n= 1 (100% 0^64^);# |
| Main caregiver (parent vs. grandparent) | n=1 (100%0^21^); # |
| Number of children or family members | n= 1 (100% 0^21^); # |
| Parent age (older) | Mother: n= 1 (100% -^64^);#  Main caregiver: n = 1 (100% 0^21^); #  Overall (main caregiver and mother): n = 2 (50%-,50% 0); # |
| Presence of Siblings | n= 1 (100% 0^59^); # |
| *Family rules and behaviours* | |
| Ambiguous tablet rules | n= 1 (100% 0^39^); # |
| Inconsistent mediation (irregular or unpredictable rules) | n= 1 (100% 0^64^); # |
| Main caregiver's perception | n= 1 (100% 0^21, 64^); # |
| Mother-child attachment instability | n= 1 (100% +^34^); # |
| Mothers' emotional intelligence | n= 1 (100% -^34^); # |
| Mother’s negative parenting behaviour | n= 1 (100% +^34^); # |
| Mother psychological aggression | n= 1 (100% 0^64^); # |
| Nonviolent discipline | n= 1 (100% 0^64^); # |
| No tablet rules | n= 1 (100% 0^64^); # |
| Parent efficacy | n= 1 (100% -^34^); # |
| Parent smartphone addiction proneness | n= 1 (100% +^38^);# |
| Parental control over children’s smartphone use | n= 1 (100% -)^38^; # |
| Parental stress | n=1 (100% +^34^); # |
| Physical assault | n= 1 (100% -^64^); # |
| Purpose of providing mobile devices to the children (tantrum; education; technology) | n= 1 (100% +^54^); # |
| Restrictive mediation (setting rules) | n= 1 (100% 0^64^); # |

Note 1 : Association codes: 0 . no association; ? . inconsistent; – . negative; + positive; # insufficient data (<3 studies) to derive an association

Note 2: If the same study provides correlations with maternal, paternal or parental, the calculation of overall association prioritises the maternal correlation first, followed by the paternal, and then the parental.
