# Supplementary material for: Correlates of mobile device use in young children: a systematic review and meta-analysis
Source: BMJ Public Health. 2026 Jun 17;4(2):e004305. doi: 10.1136/bmjph-2025-004305 (PMC13289221; doi:10.1136/bmjph-2025-004305)
Supplement: online supplemental file 8 [file bmjph-4-2-s008.docx]

**Supplementary File 8**

**Summary per device**

**Table 1.** Summary of individual and interpersonal correlates with the duration of mobile device use

Data reported per device type and across all devices, including number of studies (percentage, direction of association, study citation); overall association

| **Correlates** | **Tablet and smartphones** | **Smartphones** | **Tablets** | **Overall association** |
| --- | --- | --- | --- | --- |
| **Individual** | | | | |
| Age | n = 5 (80% +^44, 51, 55, 63^; 20% 0^56^); + | n = 6 (33.6% +^41, 66^; 16.7% -^65^; 60% 0^36, 37, 67^); 0 | n = 4 (50% +^41, 43^, 50% 0 ^36, 67^); ? | n=11 (54% +^41, 43, 44, 51, 55, 63^, - 9% - ^65^; 37% 0^36, 37, 56, 67^);? |
| Sex (boys) | n =2 (100% 0)^44, 55^; # | n= 5 (20% +^66^, 80% 0^36, 37, 45, 67^); 0 | n= 3 (100% 0^32, 36, 67^);0 | n=8 (14%+^66^, 86% 0^32, 36, 37, 45, 55, 67^ ); 0 |
| Child TV use duration | n=1 (100%+^51^); # | n=1 (100% 0^52^); # | n=2 (50%+^39^, 50% 0^52^); ? | n= 3(66.6%+^39, 51^; 33.3%0^52^; + |
| **Interpersonal** | | | | |
| *Family characteristics and structure* | | | | |
| Parental age (older) | Parental age  n= 1 (100%0^55^); #  Mother age  n = 1 (100% 0^53^); #  Spouse age  n=1 (100% +^55^); # | Mother age  n= 1 (100%+^45^); #  Father age  n=1 (100% +^45^); #  Parental age  n= 1 (100%-^58^); # | Mother age n= 1 (100% 0 ^43^); #  Parental age  n= 1 (100%-^58^); # | n= 5 (60% 0^43, 53, 55^; 20% +^45^; 20% -^58^);? |
| Parents educational level | Mother education:  n= 1^60^ (100% 0); #  Parent education:  n= 4 (50% -^51, 55^,50% 0^53, 60^); -  Spouse level education:  n= 1^55^ (100% -^55^); # | Mother education:  n= 5 (60% 0^36, 52, 66^; 40% -^45, 67^); 0  Father education:  n= 4 (25% +^66^; 25% -^45^; 50% 0^52, 67^); ?  Parent education  n= 3 (66.7% 0^36, 58^; 33.3% -^45^); 0 | Mother education:  n= 4 (25% +^52^; 75% 0^36, 43, 67^); 0  Father education  n= 2 (100% 0^52, 67^); #  Parent education:  n= 3 (33.3% -^32^; 66.7% 0^36, 58^); 0 | n=12 (33%-^32, 45, 51, 55^ ; 17% +^52, 66^ ; 50% 0^36, 43, 53, 58, 60, 67^ ); ? |
| Employment | Parents:  n= 1 (100% 0^55^); # | Mother:  n= 5 (100% 0^33, 36, 45, 52, 66^); 0  Father:  n=1 (100% 0^52^); # | Mother:  n= 2 (100% 0^36, 52^); #  Father:  n=1 (100% 0^52^); # | n= 6 (100% 0^33, 36, 45, 52, 55, 66^); 0 |
| Income | n= 3 (66.7% -^53, 55^; 33.3% 0^60^); # | n= 6 (33% -^45, 49^; 17% +^58^; 50% 0^36, 66, 67^); ? | n= 7 (28% -^39, 49^; 72% 0^32, 36, 43, 58, 67^); 0 | n=11 (36%-^39, 45, 49, 55^; 64% 0^32, 36, 43, 58, 60, 66, 67^; 0 |
| Presence of siblings | n= 2 (50% +^55^, 50% 0^53^); # | n= 1 (100% 0^67^); # | n=3 (100% 0^32, 43, 67^); # | n=5 20% +^55^; 80% 0^32, 43, 53, 67^ );0 |
| *Family rules and behaviours* | | | | |
| Parental device use | Mother’s use:  n= 1 (100% +^44, 56^); #  Father’s use:  n= 1 (100% 0^56^); #  Parent use:  n= 2 (100% +^57, 62^); +  Relative use:  n= 1 (100% +^56^); # | Parent’ use:  n= 4 (100% +^41, 58, 61, 68^); + | Mother’s use:  n= 1 (100% +^58^); #  Parent’s use:  n= 3 (100% +^41, 43, 68^); + | n= 9 (100%+^41, 43, 44, 56-58, 61, 62, 68^); + |
| Parental stress |  | n= 3 (25% +^58^; 75% 0^33, 42^); 0 | n= 1 (100% 0^58^); # | n= 3 (100% 0^33, 42, 58^);0 |
| Parental depression | n=1 (100% 0^53^); # | n= 3 (100% 0^33, 36, 58^); 0 | n= 1 (100%0^58^); # | n=4 (100% 0^33, 36, 53, 58^);0 |

Note 1: Association codes: 0 . no association; ? . inconsistent; – . negative; + positive; # insufficient data (<3 studies) to derive an association

Note 2: In the overall analysis, when studies reported data for both combined and separate devices, the combined data were prioritised. If data were provided only separately for tablets and smartphones, tablets were given priority in calculating the overall score.

Note 3: If the same study provides correlations with maternal, paternal, parental or relative, the calculation of overall association prioritises the maternal correlation first, followed by the paternal, the parental, and then the relative.
