# Supplementary material for: Correlates of mobile device use in young children: a systematic review and meta-analysis
Source: BMJ Public Health. 2026 Jun 17;4(2):e004305. doi: 10.1136/bmjph-2025-004305 (PMC13289221; doi:10.1136/bmjph-2025-004305)
Supplement: online supplemental file 9 [file bmjph-4-2-s009.docx]

**Supplementary File 9**

**Summary of the correlates of the duration of device use evidence, with less than three studies reporting an association.**

Table 1. Summary of individual correlates with the duration of mobile device use

Data reported per device type and across all devices, including number of studies (percentage, direction of association, study citation); overall association

| **Correlates** | **Tablet and smartphones** | **Smartphones** | **Tablets** | **Overall association** |
| --- | --- | --- | --- | --- |
| *Child demographics, anthropometric and health* | | | | |
| Age at the start of using tablet and smartphone | n = 1 (100% 0^56^); # | n = 1 (100% 0^45^); # |  | n=2 (100% 0^45, 56^); # |
| Age of start reading books |  | n = 1 (100% +^45^); # |  | n=1 (100% +^45^; # |
| Age at start of tablet use | n= 1 (100% 0^56^); # |  |  | n=1 (100% 0 ^56^); # |
| Gestational age (preterm or term) | n= 1 (100% 0^53^); # |  |  |  |
| BMI or BMI z-score | n= 1 (100% 0^44^); # | n= 1 (100% 0^45^); # |  | n=2 (100% 0^44, 45^); # |
| Ethnicity | n= 1 (100%0^55^); # |  | n=1 (100% +^39^); # | n=2 (50% +^39^; 50%0^55^); # |
| Medical problems child | n=2 (50%+^51^; 50% 0^55^); # |  |  | n=2 (50%+^51^; 50% 0^55^); # |
| Post natal complications | n=1 (100% 0^55^); # |  |  | n=1 (100% 0^55^); # |
| Weight | n= 1(100% 0^47^); # |  |  | n= 1(100% 0^47^); # |
| *Child behaviour and development* | | | | |
| Child book reading |  | n=1 (100% 0^52^); # | n= 2 (100% 0^32, 52^);# | n= 2 (100% 0^32, 52^);# |
| Child Lexical density |  | n=1 (100%-^52^); # | n=1 (100%+^52^);# | n=1 (100% +^52^);# |
| Child sentence use |  | n=1 (100% 0^52^); # | n=1 (100%+^52^); # | n=1 (100% +^52^);# |
| Child internalising problems |  | n=1 (100%+^58^); # | n= 1 (100% 0^58^); # | n= 1 (100% 0^58^); # |
| Child externalising problems |  | n=1 (100%+^58^); # | n= 1 (100% 0^58^); # | n= 1 (100% 0^58^); # |
| Child temperamental traits (activity level) |  | n=1 (100% 0^46^); # | n=1 (100% 0^46^); # | n=1 (100% 0^46^); # |
| Child temperamental traits (anger/frustration) |  | n=1 (100% +^46^); # | n=1 (100% 0^46^); # | n=1 (100% 0^46^); # |
| Child temperamental traits (approach) |  | n=1 (100% 0^46^); # | n=1 (100% 0^46^); # | n=1 (100% 0^46^); # |
| Child temperamental traits (attentional focusing) |  | n=1 (100% -^46^); # | n=1 (100%-^46^); # | n=1 (100% -^46^); # |
| Child temperamental traits (discomfort) |  | n=1 (100% 0^46^); # | n=1 (100% 0^46^); # | n=1 (100% 0^46^); # |
| Child temperamental traits (falling reactivity and soothability) |  | n=1 (100% -^46^); # | n=1 (100% 0^46^); # | n=1 (100% 0^46^); # |
| Child temperamental traits (fear) |  | n=1 (100% 0^46^);# | n=1 (100%-^46^);# | n=1 (100%-^46^);# |
| Child temperamental traits (high intensity pleasure) |  | n=1 (100%+^46^); # | n=1 (100% +^46^);# | n=1 (100%+^46^);# |
| Child temperamental traits (impulsivity) |  | n=1 (100% 0^46^); # | n=1 (100% 0^46^); # | n=1 (100% 0^46^); # |
| Child temperamental traits (low intensity pleasure) |  | n=1 (100% -^46^) # | n=1 (100% -^46^) # | n=1 (100%-^46^);# |
| Child temperamental traits (perceptual sensitivity) |  | n=1 (100%-^46^);# | n=1 (100%-^46^);# | n=1 (100%-^46^);# |
| Child temperamental traits (sadness) |  | n=1 (100%-^46^);# | n=1 (100%-^46^);# | n=1 (100%-^46^);# |
| Child temperamental traits (shyness) |  | n=1 (100%0^46^);# | n=1 (100%-^46^);# | n=1 (100%-^46^);# |
| Child temperamental traits (smiling and laughter) |  | n=1 (100%0^46^);# | n=1 (100%0^46^);# | n=1 (100%0^46^);# |
| Children’s outdoor activity |  | n= 1 (100% -^53^); # |  | n= 1 (100% -^53^); # |
| Emergent meta cognition Index | n=1 (100% 0^40^); # |  |  | n=1 (100% 0^40^); # |
| Flexibility index | n=1 (100% 0^40^); # |  |  | n=1 (100% 0^40^); # |
| General Executive Composite | n=1 (100% 0^40^); # |  |  | n=1 (100% 0^40^); # |
| Inhibitory self-control | n=1 (100% 0^40^); # | n=1 (100% -^46^); # | n= 1 (100% -^46^); # | n=2 (50% -^46^; 50% 0^40^); # |
| Language milestones |  | n=1 (100% 0^52^); # | n=1 (100% 0^52^); # | n=1 (100% 0^52^); # |
| Motor skills milestones |  | n=1 (100% 0^52^); # | n=1 (100% 0^52^); # | n=1 (100% 0^52^); # |
| Nutritional score | n=1 (100%+^47^); # |  |  | n=1 (100%+^47^); # |
| Sleep problems |  | n=1(100% 0^45^); # |  | n=1(100% 0^45^); # |
| Watch TV (y/n) | n=1 (100%+^51^);# |  |  | n= 1(100%+^51^); # |

Note 1: Association codes: 0 . no association; ? . inconsistent; – . negative; + positive; # insufficient data (<3 studies) to derive an association

Note 2: In the overall analysis, when studies reported data for both combined and separate devices, the combined data were prioritised. If data were provided only separately for tablets and smartphones, tablets were given priority in calculating the overall score.

Table 2. Summary of interpersonal correlates with the duration of mobile device use

Data reported per device type and across all devices, including number of studies (percentage, direction of association, study citation); overall association

Interpersonal

| **Correlates** | **Tablet and smartphones** | **Smartphones** | **Tablets** | **Overall association** |
| --- | --- | --- | --- | --- |
| *Family characteristics and structure* |  |  |  |  |
| Birth order | n= 1 (100% +^55^); # |  |  | n= 1 (100% +^55^); # |
| Family type (nucleus vs extended) | n= 1 (100%0^55^); # |  |  | n= 1 (100%0^55^); # |
| Main caregiver (parent vs. grandparent) | n= 1 (100%0^55^); # |  |  | n= 1 (100%0^55^); # |
| Marriage status | n= 1 (100%0^55^); # |  |  | n= 1 (100%0^55^); # |
| Parent BMI | Maternal BMI  n= 1 (100%+^53^); #  Paternal BMI  n=1 (100% +^53^); # |  |  | n= 1 (100%+^53^); # |
| Medical problem parent | n= 1 (100%0^55^); # |  |  | n= 1 (100%0^55^); # |
| Number of children or family members | n= 1 (100% 0^55^); # | n= 1 (100%-^66^);# |  | n= 2 (50% -^66^; 50%0^55^); # |
| Parents working hours |  | n= 1 (100%+^51^); # |  | n= 1 (100%+^51^); # |
| *Family rules and behaviours* |  |  |  |  |
| Adult smartphone addiction |  | n= 1 (100%0^33^); # |  | n= 1 (100%0^33^); # |
| Ambiguous tablet rules |  |  | n= 1 (100%0^39^); # | n= 1 (100%0^39^); # |
| Background TV |  | n= 2 (100%0^31, 52^); # | n= 2 (100%0^31, 52^); # | n= 2 (100%0^31, 52^); # |
| Frequency Parent read child |  |  | n= 1 (100%-^32^); # | n= 1 (100%-^32^); # |
| Internet parenting style scale (parental control) |  | n=1 (100% -^48^); # |  | n=1 (100% -^48^); # |
| Internet parenting style scale (parental warmth) |  | n=1 (100% 0^48^); # |  | n=1 (100% 0^48^); # |
| No tablet rules |  |  | n= 1 (100%0^39^); # | n= 1 (100%0^39^); # |
| Parent attitudes/ behaviour towards media/technology |  | n= 1 (100%+^41^); # | n= 1 (100%+^41^); # | n= 1 (100%+^41^); # |
| Parent efficacy |  | n= 1 (100%-^37^); # | n= 1 (100%-^32^); # | n= 2 (100% -^32, 37^); # |
| Parent seeing as a benefit for the child | n=1 (100% 0^63^); # |  |  | n=1 (100% 0^63^); # |
| Parents seeing as a benefit for parents | n=1 (100% +^63^); # |  |  | n=1 (100% +^63^); # |
| Parental TV time |  | n= 1 (100%+^45^); # |  | n= 1 (100%+^45^); # |
| Parental internet time |  | n= 1 (100%+^45^); # |  | n= 1 (100%+^45^); # |
| Parental knowledge about smartphones exposure |  | n= 1 (100%-^61^); # |  | n= 1 (100%-^61^); # |
| Parental attitudes (excessive motherhood) |  | n= 1 (100%+^46^); # | n= 1 (100%0^46^); # | n= 1 (100%0^46^); # |
| Parental attitudes (democratic attitude and recognition of equality) | n= 1 (100%0^48^); # | n= 1 (100%0^46^); # | n= 1 (100%0^46^); # | n= 2 (100%0^46, 48^); # |
| Parental attitudes (hostile and rejective attitude) |  | n= 1 (100%0^46^); # | n= 1 (100%0^46^); # | n= 1 (100%0^46^); # |
| Parental attitudes (discord between parents) |  | n= 1 (100%+^46^); # | n= 1 (100%+^46^); # | n= 1 (100%+^46^); # |
| Parental attitudes (authoritarian attitude) | n= 1 (100%0^48^); # | n= 1 (100%+^46^); # | n= 1 (100%+^46^); # | n= 2 (50%+^46^, 50% 0^48^); # |
| Parental attitudes (tolerant) | n= 1 (100%0^48^); # |  |  | n= 1 (100%0^48^); # |
| Parental mediation strategies (Restrictive Strategy) | n= 2 (50%-^44^ 50%0^63^); # |  |  | n= 2 (50%-^44^ 50%0^63^); # |
| Parental mediation strategies (Active Strategy) | n= 1 (100%+^63^); # |  |  | n= 1 (100%+^63^); # |
| Parental mediation strategies (Co-use Strategy) | n= 2 (50%+^44^ ;50%0^63^); # |  |  | n= 2 (50%+^44^ ;50%0^63^); # |
| Parental mediation strategies (Technical safety ) | n= 1 (100%0^63^); # |  |  | n= 1 (100%0^63^); # |
| Parental mediation strategies (Diversionary) | n= 1 (100%+^63^); # |  |  | n= 1 (100%+^63^); # |
| Parental anxiety |  | n= 2 (50%+^58^; 50%0^33^); # | n= 1 (100%+^58^); # | n=2 (50% +^58^; 50%0^33^; # |
| Percentage of time the child spends alone on smartphone |  | n= 1 (100%+^65^); # |  | n= 1 (100%+^65^); # |
| Personal wellbeing mother |  |  | n= 1 (100%-^43^); # | n= 1 (100%-^43^); # |
| Relational wellbeing mother (relationship satisfaction) |  |  | n= 1 (100%-^43^); # | n= 1 (100%-^43^); # |
| Screen-related parenting practices (behaviour regulation) | n= 1 (100%+^44^); # |  |  | n= 1 (100%+^44^); # |
| Screen restricted content | n= 1 (100%-^44^); # |  |  | n= 1 (100%-^44^); # |
| Share TV with adult |  | n= 1 (100%0^52^); # | n= 1 (100%+^52^); # | n= 1 (100%+^52^); # |
| Share cell phone with adult |  | n= 1 (100%0^52^); # | n= 1 (100%0^52^); # | n= 1 (100%0^52^); # |
| Share PC with adult |  | n= 1 (100%0^52^); # | n= 1 (100%0^52^); # | n= 1 (100%0^52^); # |
| Share tablet with adult |  | n= 1 (100%0^52^); # | n= 1 (100%0^52^); # | n= 1 (100%0^52^); # |
| Share internet with adult |  | n= 1 (100%0^52^); # | n= 1 (100%0^52^); # | n= 1 (100%0^52^); # |
| Spent time with children |  | n= 1 (100%-^55^); # |  | n= 1 (100%-^55^); # |

Note 1: Association codes: 0 . no association; ? . inconsistent; – . negative; + positive; # insufficient data (<3 studies) to derive an association

Note 2: In the overall analysis, when studies reported data for both combined and separate devices, the combined data were prioritised. If data were provided only separately for tablets and smartphones, tablets were given priority in calculating the overall score.

Note 3: If the same study provides correlations with maternal, paternal, parental or relative, the calculation of overall association prioritises the maternal correlation first, followed by the paternal, the parental, and then the relative.

Table 3. Summary of environmental correlates with the duration of mobile device use.

Data reported per device type and across all devices, including number of studies (percentage, direction of association, study citation); overall association.

| **Correlates** | **Tablet and smartphones** | **Smartphones** | **Tablets** | **Overall association** |
| --- | --- | --- | --- | --- |
| Criminal rates of the neighbourhood |  | Boys: n= 1 (100%0^31^); #  Girls: n= 1 (100%+^31^); # | Boys: n= 1 (100%0^31^); #  Girls: n= 1 (100%+^31^); # | Boys: n= 1 (100%0^31^); #  Girls: n= 1 (100%+^31^); # |
| Good environment of the neighbourhood to practice physical activity |  | Boys: n= 1 (100%0^31^); #  Girls: n= 1 (100%0^31^); # | Boys: n= 1 (100%0^31^); #  Girls: n= 1 (100%0^31^); # | Boys: n= 1 (100%0^31^); #  Girls: n= 1 (100%0^31^); # |
| Good environment of the neighbourhood to walk |  | Boys: n= 1 (100%0^31^); #  Girls: n= 1 (100%0^31^); # | Boys: n= 1 (100%0^31^); #  Girls: n= 1 (100%0^31^); # | Boys: n= 1 (100%0^31^); #  Girls: n= 1 (100%0^31^); # |
| Traffic level of the neighbourhood |  | Boys: n= 1 (100%0^31^); #  Girls: n= 1 (100%0^31^); # | Boys: n= 1 (100%0^31^); #  Girls: n= 1 (100%0^31^); # | Boys: n= 1 (100%0^31^); #  Girls: n= 1 (100%0^31^); # |

Note 1: Association codes: 0 . no association; ? . inconsistent; – . negative; + positive; # insufficient data (<3 studies) to derive an association

Note 2: In the overall analysis, when studies reported data for both combined and separate devices, the combined data were prioritised. If data were provided only separately for tablets and smartphones, tablets were given priority in calculating the overall score.
