# Supplementary material for: Correlates of mobile device use in young children: a systematic review and meta-analysis
Source: BMJ Public Health. 2026 Jun 17;4(2):e004305. doi: 10.1136/bmjph-2025-004305 (PMC13289221; doi:10.1136/bmjph-2025-004305)
Supplement: online supplemental file 10 [file bmjph-4-2-s010.docx]

**Supplementary File 10.** Meta-analyses of correlates of problematic smartphone use and the duration of mobile device use

| Correlate | Transformed effect^1^ | 95% CI^2^ | Z-statistic for transformed effect | P-value for transformed effect | I^2^ statistic | Effect^3^ | 95% CI^4^ |
| --- | --- | --- | --- | --- | --- | --- | --- |
| **Problematic Smartphone Use** | | | | | | | |
| Child age  n=4^21, 54, 59, 64^ | 0.206 | (-0.0300, 0.421) | 1.71 | 0.087 | 98.7% | 0.203 | (-0.0300, 0.398) |
| Child gender n=3^21, 54, 64^ | -0.117 | (-0.358, 0.118) | 0.898 | 0.368 | 98.2% | -0.116 | (-0.343, 0.117) |
| Parental device use n=3^34, 35, 59^ | 0.149 | (-0.109, 0.386) | 1.136 | 0.256 | 98.5% | 0.148 | (-0.109, 0.368) |
| Parents educational level  n=3^54, 59, 64^ | 0.0729 | (-0.404, 0.508) | 0.287 | 0.774 | 99.4% | 0.0728 | (-0.383, 0.468) |
| Family income n=4 ^21, 39, 54, 59^ | 0.0759 | (-0.115, 0.260) | 0.778 | 0.436 | 98.3% | 0.0758 | (-0.114, 0.254) |
| **Duration of Mobile Device Use** | | | | | | | |
| Age (tablet)  n=4 ^37, 41, 66, 67^ | 0.211 | (0.006, 0.416) | 2.02 | 0.044 | 98.0% | 0.208 | (0.006, 0.394) |
| Age (Smartphone)  n=4 ^37, 41, 44, 66^ | 0.107 | (0.035, 0.179) | 2.91 | 0.004 | 61.7% | 0.107 | (0.035, 0.177) |
| Age (combined)  n=4 ^51, 55, 56, 63^ | 0.182 | (0.063, 0.302) | 2.98 | 0.003 | 63.6% | 0.180 | (0.063, 0.293) |
| Sex (smartphone)  n=4^37, 45, 65, 66^ | 0.051 | (0.010, 0.101) | 1.98 | 0.047 | 0.0% | 0.051 | (0.010, 0.101) |
| Parental device use (tablet)  n=4 ^41, 43, 58, 68^ | 0.417 | (0.290, 0.544) | 6.44 | <0.001 | 76.1% | 0.394 | (0.282, 0.486) |
| Parental device use (smartphone)  n=5 ^41, 57, 58, 61, 68^ | 0.237 | (0.134, 0.340) | 4.51 | <0.001 | 91.2% | 0.233 | (0.133, 0.327) |
| Parents educational level (tablet)  n=3 ^43, 52, 58^ | 0.028 | (-0.152, 0.208) | 0.304 | 0.761 | 94.8% | 0.028 | (-0.151, 0.205) |
| Parents educational level (smartphone)  n=6 ^45, 52, 58, 61, 65, 66^ | -0.022 | (-0.119, 0.075) | -0.447 | 0.655 | 84.7% | -0.022 | (-0.119, 0.075) |
| Parents educational level (combined)  n=4 ^32, 51, 52, 55^ | -0.035 | (-0.088, 0.107) | -1.33 | 0.183 | 35.0% | -0.035 | (-0.088, 0.107) |
| Employment (smartphone) n=4 ^33, 45, 52, 66^ | 0.020 | (-0.032, 0.072) | 0.753 | 0.452 | 0.0% | 0.020 | (-0.032, 0.072) |
| Income (tablet) n=5 ^32, 39, 43, 49, 58^ | 0.020 | (-0.054, 0.093) | 0.520 | 0.603 | 90.7% | 0.021 | (-0.054, 0.093) |
| Income (smartphone)  n=4 ^45, 49, 58, 66^ | -0.008 | (-0.081, 0.065) | -0.213 | 0.832 | 70.6% | 0.006 | (-0.081, 0.065) |
| Parental stress (smartphone) n=3^33, 42, 58^ | 0.168 | (0.065, 0.271) | 3.18 | 0.001 | 49.3% | 0.166 | (0.065, 0.265) |
| Parental age (smartphone) n=3^45, 55, 58^ | -0.034 | (-0.210, 0.142) | -0.381 | 0.703 | 88.7% | -0.034 | (-0.207, 0.141) |

^1^Fisher transform of the correlation coefficient or phi statistic; ^2^Calculated from Fisher transform of effect size; ^3^Back-transformed effect (correlation coefficient or phi statistic); ^4^Back-transformed confidence interval for effect.

Note1: The reference category of all ordinal categorical variables is the lowest level of the variable (e.g. no formal education; unemployed). The reference category of the sex variable is male.

Note 2: For PSU – Parents’ educational level, we prioritised maternal data over paternal in study 54 – Abdullah et al. 2022. Similarly, for Mobile device use, we prioritised maternal data over paternal for the correlates: 1) Parents’ educational level, studies 52 and 66 - Gago-Galvano 2023 and Rathnasiri et al., 2022, respectively and 2) Employment (smartphone), study 52 - Gago-Galvano 2023.
